# Gene Networks of Fully Connected Triads with Complete Auto-Activation Enable Multistability and Stepwise Stochastic Transitions

**DOI:** 10.1371/journal.pone.0102873

**Published:** 2014-07-24

**Authors:** Philippe C. Faucon, Keith Pardee, Roshan M. Kumar, Hu Li, Yuin-Han Loh, Xiao Wang

**Affiliations:** 1 School of Computing, Informatics, Decision Systems Engineering, Arizona State University, Tempe, Arizona, United States of America; 2 Wyss Institute for Biological Inspired Engineering, Harvard University, Boston, Massachusetts, United States of America; 3 Center for BioDynamics and Center for Advanced Biotechnology, Boston University, Boston, Massachusetts, United States of America; 4 Department of Molecular Pharmacology and Experimental Therapeutics, Center for Individualized Medicine, Mayo Clinic, Rochester, Minnesota, United States of America; 5 Epigenetics and Cell Fates Laboratory, A*STAR Institute of Molecular and Cell Biology, Department of Biological Sciences, National University of Singapore, Singapore, Singapore; 6 School of Biological and Health Systems Engineering, Arizona State University, Tempe, Arizona, United States of America; University of Southampton, United Kingdom

## Abstract

Fully-connected triads (FCTs), such as the Oct4-Sox2-Nanog triad, have been implicated as recurring transcriptional motifs embedded within the regulatory networks that specify and maintain cellular states. To explore the possible connections between FCT topologies and cell fate determinations, we employed computational network screening to search all possible FCT topologies for multistability, a dynamic property that allows the rise of alternate regulatory states from the same transcriptional network. The search yielded a hierarchy of FCTs with various potentials for multistability, including several topologies capable of reaching eight distinct stable states. Our analyses suggested that complete auto-activation is an effective indicator for multistability, and, when gene expression noise was incorporated into the model, the networks were able to transit multiple states spontaneously. Different levels of stochasticity were found to either induce or disrupt random state transitioning with some transitions requiring layovers at one or more intermediate states. Using this framework we simulated a simplified model of induced pluripotency by including constitutive overexpression terms. The corresponding FCT showed random state transitioning from a terminal state to the pluripotent state, with the temporal distribution of this transition matching published experimental data. This work establishes a potential theoretical framework for understanding cell fate determinations by connecting conserved regulatory modules with network dynamics. Our results could also be employed experimentally, using established developmental transcription factors as seeds, to locate cell lineage specification networks by using auto-activation as a cipher.

## Introduction

Embryonic stem cells have the capacity to differentiate into more than 200 isogenic progenitor and terminal cell types [1], each of which represents a stable cellular state. The potential for a system to realize multiple states is termed multistability, which has been proposed as the mechanism underlying cell differentiation for over half a century, with stable states represented as either valleys in the developmental landscape [2], [3] or as dynamic attractors in high-dimensional gene expression space [4]–[6]. Bistability is the simplest form of multistability, which has been studied extensively both in natural contexts [7] and in synthetic systems [8]–[13]. It has been shown to be responsible for both normal cell fate determination in *Xenopus* oocyte maturation [14] and oncogenesis [15]. Additionally, recent work in hematopoietic stem cells (HSC) implies that more complicated cases of multistability help in canalizing HSC lineage determination [16]. The discovery of induced pluripotent stem cells (iPSC) [17]–[20] further demonstrates that cellular state transitioning within a multistable system is a reversible process that can potentially be engineered for therapeutic purposes.

**Figure 1:**
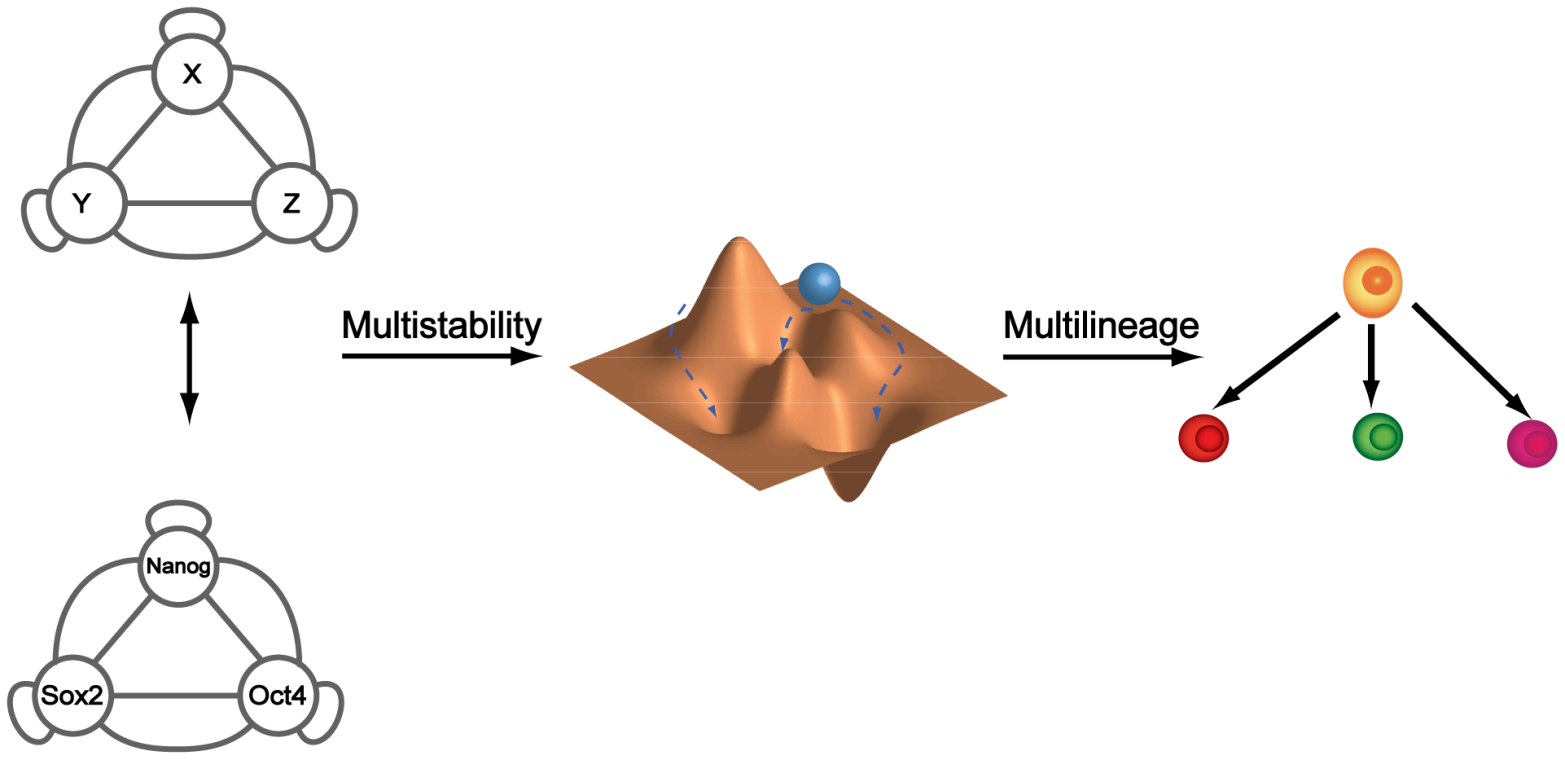
Multistability arises from small gene networks and underlies cell differentiation. Fully-connected triads (FCTs) are important, recurring transcriptional networks in the development and maintenance of cellular states. Notably, the Oct4-Sox2-Nanog triad has been implicated in inducing and maintaining stem cell pluripotency. In the Waddington model for cell differentiation [3], the cell's underlying developmental landscape is governed by the dynamical potential of these small gene networks to realize multistability. Transition from one state to another can be guided via altered topology or strength of wiring.

Despite evidence for the existence of multiple substates of pluripotent and hematopoietic stem cells [21]–[25], the molecular mechanisms responsible for generating complex multistability are poorly understood. Transitions between stable states may underlie widely observed stem cell population heterogeneity [23], [25]–[34], random latency in induced pluripotency [34], [35], and even the progression of diseases such as cancer [36], [37]. According to this view, regulatory networks capable of adopting multiple stable states may enable cells to explore adjacent fate possibilities through state transitions, thereby enabling differentiation in the case of stem cells, or generating phenotypic diversity for selection to act upon in the case of tumor evolution. Recent studies have implicated the fully-connected triad (FCT) [38]–[40] as a recurring network motif among the transcriptional regulatory circuits that control the development and maintenance of cellular states. Most notably, the Oct4-Sox2-Nanog triad is believed to be the key functional module in the maintenance of stem cell pluripotency [41]–[43]. The success of induced pluripotency through the overexpression of transcription factors alone further indicates the dominant role of transcription factor networks in maintaining pluripotency and directing cellular reprogramming. All this suggests a strong relationship between biological multistability and the conserved FCT network of transcription factors (Figure 1), and motivates a need for understanding the relationship between FCT regulatory relationships and multistability.

Here we developed a high-throughput computational approach to search the network dynamics of all fully-connected transcription triads for their potential to realize multistability. Our computational searches identified complete auto-activation, (i.e.: all three nodes activate themselves), as a topological feature of triads capable of multiple stable steady states (SSS). Detailed analyses were performed on network topologies selected for a high likelihood of multistability. These analyses show that the relative stability of SSS can be tuned by adjusting the network regulatory strengths between nodes. After incorporation of gene expression noise in stochastic simulations, the system was able to spontaneously transit from metastable (SSS with relatively lower stabilities) to differentiated states (SSS with at least one gene OFF and high stabilities), behaving like a three-dimensional Markovian jump system with bias. This is also consistent with experimentally demonstrated stochastic cancer cell state transitions [44], which result in a phenotypic equilibrium in populations of cancer cells.

Different levels of noise, designed to mimic degrees of “noisy” transcriptional activity in cellular systems, were found to either promote or disrupt state transitions, with some transitions requiring layovers at one or more intermediate states. Such connections between gene expression noise and cell state transitions have also been demonstrated in many biological processes [45]. Finally, in our simplified theoretical simulation of reprogramming to generate induced pluripotent stem cells, the distribution of random temporal latency could be described by an Inverse Gaussian distribution, a finding consistent with experimental observations [34]. Taken together, our work illustrates that computational screening, analysis, and simulation of network dynamics can serve as valuable tools for connecting conserved regulatory modules with complex biological processes, such as development, disease and stem cell reprogramming.

## Results

### Network Enumeration and Parameter Scanning for Multistability

**Figure 2:**
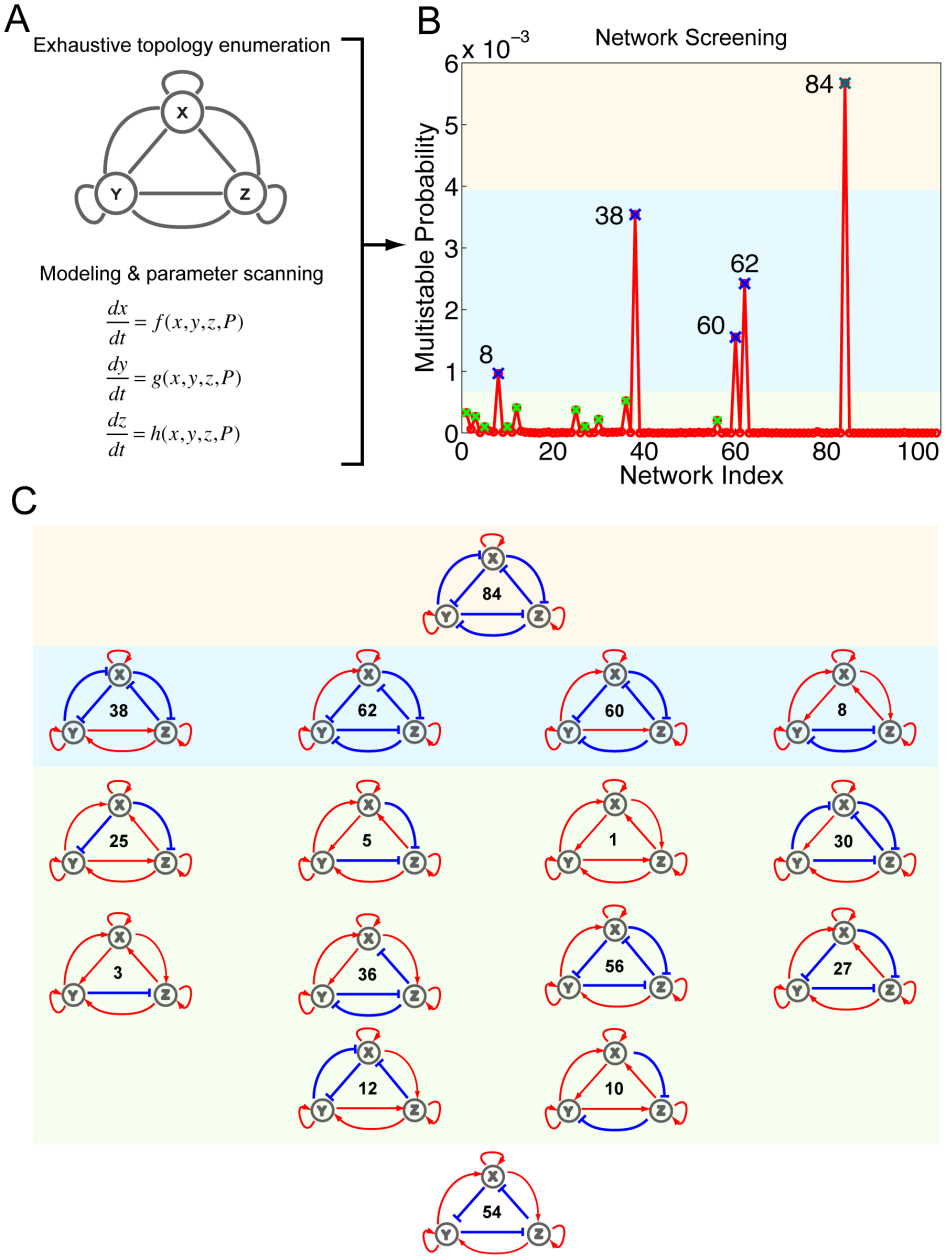
High-throughput computational screening for multistable networks. (A) FCTs were enumerated and modeled by ODEs (parameters denoted as *P*). (B) The probability of multistability for each of the 104 FCTs generated by the computational screen is plotted. FCTs with a high probability of multistability are marked with an X with the color representing the multistability group; the two highest probability groups are labeled by their indices. Colored backgrounds serve as visual aids to distinguish the top three groups. (C) Fifteen FCTs with significant multistable potentials are listed with groups ranked according to their probability for multistability. The red arrows indicate activating regulation and blue T-head arrows indicate inhibitory regulation. Network 54 was included because it was the only network with complete auto-activation that did not have a high multistable potential.

To define the set of FCT networks with a high likelihood for multistability, we first enumerated all possible fully-connected three-node topologies (Figure 2A). Here, nodes represent transcription factors and edges represent possible transcriptional regulations. After taking into account topological equivalencies, we were left with 104 unique FCTs as candidate topologies, which fully represent all 2^9^ = 512 possible FCT topologies (see Supporting Information (SI), Figure S1 and MATERIALS AND METHODS for details).

Each FCT candidate was generally modeled by a set of three-variable ordinary differential equations (ODEs), with each variable representing the abundance of a single transcription factor and each of the nine tunable parameters representing a single regulation strength (Figure 2A). The activation and repression of each gene by other factors are modeled additively to account for reported mechanistic independence of transcription regulations [16], [40], [46] (MATERIALS AND METHODS). This simplification can be expanded to include more mechanistic details of specific networks without altering the general conclusion of this screening. A state of the network is then defined as an equilibrium or SSS if all transcription factor abundances do not change over time. An algorithm was developed to automate the search process to identify all possible states.

Specifically, to measure the probability of multistability, we scanned N = 421,875 parameter sets for each of the 104 networks to identify all possible states. The values of these parameters are generic but cover a wide range to serve as a proof of principle for diverse biological scenarios. The probability of multistability was then defined as the number of parameter sets giving rise to at least 5 stable steady states divided by N. This probability reflects the robustness of certain FCT's multistability, and hence quantifies its possible natural presence frequency. The entire FCT multistability screen is an ensemble of many independent computations and, as a result, was run in parallel and performed in high-throughput computer clusters (see MATERIALS AND METHODS). Such large-scale screening is computationally intensive and is only possible with simplified model equations. Therefore the chosen forms of our ODE models omit many biochemical details such as epigenetics and transcriptional factor co-binding. The models serve as a general abstraction and system specific details could be incorporated later in light of specific experimental evidence.

### Complete Auto-Activation Contributes to Multistability

Using our network screening approach, we quantified and ranked the potential for multistability of all 104 possible FCTs (Figure 2A). Of the 104, 15 showed significant probability for multistability (marked in Figure 2B and topologies drawn in Figure 2C).

The multistable FCTs were empirically categorized into three groups according to their probability of multistability (color shaded in Figure 2B,C). The first group, which had the highest probability of realizing multistability and with the potential for eight states, consisted of a single network (Network 84) with all positive auto-regulation and all mutual inhibition. The second group of multistable FCTs, consisting of Networks 8, 38, 60, and 62, was also exclusively positive auto-regulating but displayed a mixture of positive and negative mutual regulation. Specifically, these networks were either enriched for mutual inhibition (Networks 60 and 62) or displayed symmetrical topologies (Network 8 and 38). The third group, which was comprised of 10 FCTs that exhibited lower but still significant potential for multistability, once again featured similar positive auto-regulation as a common attribute. The networks of the third group had either asymmetric topologies (Networks 3, 10, 12, 27, 36 and 56) or competing mutual regulation of target genes (Network 5, 25, and 30), except for Network 1, which was entirely void of inhibitory regulation. These results suggest that an appropriate combination of mutual inhibitory and activating regulation helps to increase the potential for a network to realize multistability, a finding that is consistent with previous theoretical studies [47].

From our screen, we were able to identify a set of important, minimum topological features capable of multistability. In particular, of the possible 16 FCTs with complete positive auto-regulation, our screen selected 15 as having a high likelihood for multistability. Network 54 was the only FCT displaying complete auto-activation with insignificant potential for multistability, likely because of competing mutual activation and repression of all the nodes (bottom of Figure 2C).

Indeed, positive auto-regulation has been shown to be an important feature of bistable systems [7], [9], [10], [14], [48], [49], and recent experimental work also suggests it could be necessary for maintaining multipotency in stem cells [50]. The role of positive feedback in multistability has been well studied [9], [14], [51], and the interconnected autoregulatory loop formed by Oct4, Sox2, and Nanog is thought to underlie the bistable switch between ESC self-renewal and differentiation, and the ability of core ESC regulators to ‘boot up’ the pluripotency transcriptional program by forced expression during reprogramming [52]. Our computational results similarly suggest that, within the limits of the study, complete positive auto-regulation is an important contributor to the generation of high-dimensional multistability across diverse FCT topologies. It is striking to note that one or two instances of auto-activation, no matter how strong, are not sufficient to generate multistability in our simulations. Therefore, it is possible that complete auto-activation of FCTs could be used as an important hallmark in the identification of new multistable gene networks.

### Sensitivity and bifurcation of multistability

**Figure 3:**
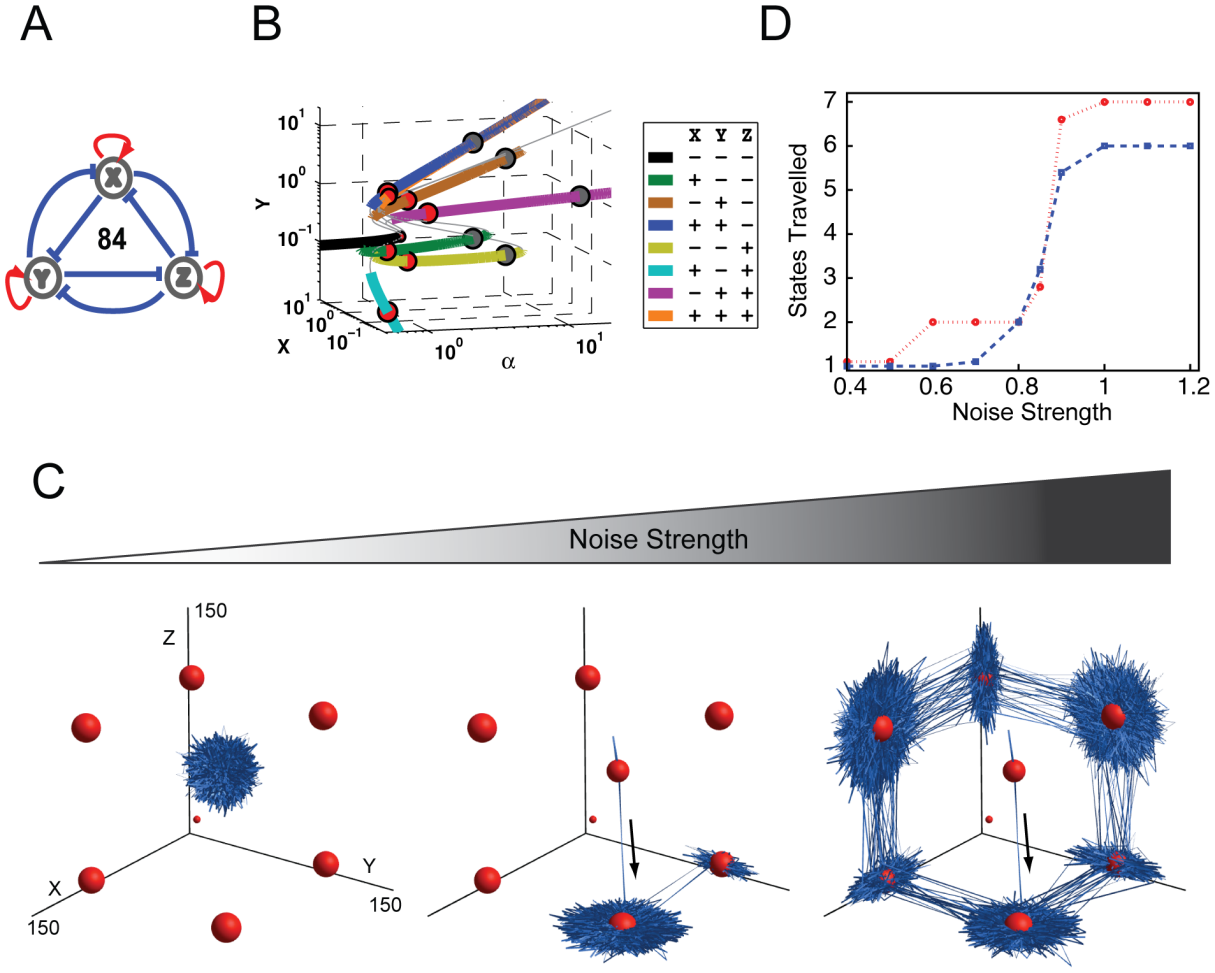
Bifurcation and Stochastic Analysis of Network 84. (A) The topology of Network 84 was predicted to have the highest probability for multistability. (B) The bifurcation diagram of Network 84 plots transcription factor concentrations of X and Y at each SSS against α, the self-activation strength. The mutual inhibition parameters are also set equal and referred to as β. Here β is set to 0.1 and α is used as the bifurcation parameter ranging between .01 and 100. By including both stable (colored) and unstable (grey) steady states we can see that as α increases the SSS move to higher concentrations, approximately at the same rate as the increase in α. SSS are color coded and listed in the legend to distinguish different SSS, where ‘+’ indicates the gene is ‘ON’ and ‘-’ means ‘OFF’. Spheres are also attached to each SSS at α = 1 (red) and α = 5 (gray), sphere size correlates with the size of the spectral radius, a measure of SSS stability. (C) Simulations of noise-induced state transitioning in Network 84 under different levels of noise. Simulations were performed with auto-regulation equal to 1 and mutual inhibition equal to 0.1, with noise levels of 0.5, 0.85, and 1 from left to right. The locations of the deterministically calculated states are indicated with red spheres, with their size correlated to their spectral radius. The blue ribbons indicate the temporal trajectories of the simulations. The black arrows indicate initial direction of state transitions. Potential cloud shape for each SSS is illustrated in Figure S6A. (D) The number of states traveled in the stochastic simulations plotted versus noise strength. The red line represents simulations that were initiated from the all-ON state, and the blue line represents simulations that were initiated from the origin.

Network 84 (Figure 3A, all positive auto-regulation and all mutual inhibition) yielded the highest potential for multistability in our screen (Figure 2C). To gain a deeper understanding of this FCT, we performed a bifurcation analysis [53], [54] to investigate how the change of one regulation strength affects the number, stability, and location of all states, while other parameters are kept constant (Figures 3B and S2). Bifurcation analysis is a useful analytical tool that can reveal when and where one state can bifurcate into two or more states in response to a changing parameter. This is analogous to a progenitor cell transitioning from a self-renewing phase to a multi-lineage differentiating phase in response to a stimulus [16].

In the bifurcation diagram of Network 84 (Figures 3B and S2), locations of both SSS (thick colored lines) and unstable steady states (thin grayed lines) are plotted. X and Y indicate the abundance of gene product of X and Y. The axis of α represents the strength of all self-activation, while other parameters are kept constant. Gene product Z is omitted because only three quantities can be visualized in the plot. Nevertheless, its abundance can be inferred from that of X and Y. Thus the figure provides the position of SSS as the concentration of the three transcription factors, and their self-activation strength, varies.

As can be seen in Figure 3B, Network 84 has complex dynamics. When auto-activation is very weak (α less than 1), the system only has one SSS (the black line), which corresponds to the state in which the abundances of all three gene products are very low and could be roughly categorized as all genes OFF. This finding intuitively makes sense considering the mutual repression of Network 84's topology, which under weak auto-activation does not support individual genes overcoming degradation to support the ON state. However, as the strengths of auto-activation increase beyond a threshold, the system suddenly demonstrates many more SSS (colored solid lines). All of these SSS branch out from the all-OFF state, which is the typical nature of bifurcation. When α = 1, the system can assume its maximum of eight states (illustrated in Figure 3B as red circles). These states include the following possible binary combinations: all three genes ON (orange); all three genes OFF (black); two genes ON and one gene OFF (blue, cyan, and purple); and, one gene ON and two genes OFF (green, brown, and yellow). The locations of these eight SSS are also plotted in Figure 3C in XYZ coordinate. The sizes of the circles correspond to each state's stability (details below). Network 84 is able to maintain multiple SSS for a wide parameter range of auto-activation. The all-OFF state (Figure 3B black line) disappears when auto-activation strengths become greater than 1.05 (Figure 3B gray circles illustrate locations of SSS when α = 5). As the auto-activations become stronger (e.g., α greater than 7), all three one-gene-ON (green, brown, and yellow) states disappeared, leaving only two-gene-ON and all-ON states to be SSS (blue, cyan, purple, and orange lines in Figure 3B).

The potential for this three-node gene network to yield as many as eight stable states underscores the idea that many cell types (e.g., over 200) could be generated from networks comprised of a small number of nodes. Taken further, a fully-connected network with only eight nodes could produce 256 states [47]. While only theoretical, it is an example of the potential for such combinatorial complexity and provides a plausible mechanism for cell lineage determination, where different gene product abundances and ON-OFF combinations could drive the system towards a spectrum of cell fates. Coupled with our bifurcation analysis, where we modeled the abrupt appearance or collapse of SSS in this topology, FCTs could also lead to discrete jumps between states in response to a changing cellular environment or signaling. The phenomenon of hysteresis, when such jumps cannot be reversed by returning to the previous environmental conditions, has been established as the mechanism for many irreversible cell fate determinations[55].

### Stochastic State Transitioning

Next, we investigated the effects of gene expression fluctuations on the multistability potential of Network 84. Fluctuations in gene expression under constant environmental conditions may arise from inherent stochastic fluctuations in gene expression, or from regulatory architecture, chromatin structure, or transcriptional bursting. Here, we use the broad term ‘gene expression noise’ to encompass all these possibilities. Gene expression noise has been shown to affect cellular physiology in single-cell organisms [56]–[62], and has also been linked to random developmental patterns and cell fate decisions in multicellular organisms [63]–[67]. The mechanisms underlying the utilization and regulation of gene expression noise in higher organisms, however, are not well understood. We introduced such noise into our model by modifying the algorithm from Adalsteinsson et al. [68] to help understand how inherent stochasticity affects cell differentiation (see MATERIALS AND METHODS).

Using our modified algorithm, we simulated the temporal evolution of all three gene product abundances under the influence of different levels of gene expression noise. The sum of these calculated measures can be visualized as the time-lapse movie of a FCT's fate over time (Movie S1). The system was initialized at the all-ON state to mimic the promiscuous gene expression state that is often observed when various progenitor cells start to differentiate [69]–[73]. The locations of eight SSS when α = 1 are indicated by the red spheres in Figure 3C, where the size of the spheres represents the stability of each state as measured by its spectral radius [74], [75]. As shown in Figure 3C, at small noise levels (Figure 3C, left panel), expression of all three genes displayed fluctuations, yet the trajectory remained focused around the initial all-ON state, indicating that small levels of gene expression noise were not sufficient to induce spontaneous state transitioning in Network 84. This also indicates the stability of the network under well-buffered conditions. As the level of noise was increased to an intermediate level (Figure 3C, middle panel), the system began to transition from the initial all-ON state to a two-ON-one-OFF state and later to a two-OFF-one-ON state. Within the time frame simulated, the system transitioned back and forth between these two latter states and spent most of the time in the vicinity of these two states. When the noise level was increased further, the system was able to visit all six states with at least one gene ON and one gene OFF (Figure 3C, right panel; Movie S1).

The results of the stochastic state-transition simulations have several interesting biological implications. First, the system transitioned exclusively between states with at least one gene ON and one gene OFF. After jumping out of the initial state, it never returned to the all-ON state within the simulation time frame; moreover, it never reached the all-OFF state. For this topology, the all-ON and all-OFF states are significantly less stable than the other six states, and thus serve as weaker attractors. Specifically, it is the fine balance in the abundance of all gene products that determines the stability of the all-ON and all-OFF states, and this balance is easily disrupted by random perturbations such as gene expression noise. These results provide a simplified and yet concrete demonstration of the recently proposed concept of “dynamic equilibrium” [76], in which individual cells can jump between states randomly, while the proportion of cells in a population at one specific state remains constant. Here we can see the proportion (probability) of cells in one state is determined by the stability of each state, which in turn is determined by network topology and system parameters. Therefore when one single fluorescence reporter tagged to one gene is experimentally used to track single cell dynamics, the cell population will exhibit a broad distribution because this multi-dimensional system is projected onto a single measured dimension.

Second, state transitions were only observed between two adjacent states and not between non-neighboring states. In other words, the transition from one state to another non-neighboring state required layovers at one or more intermediate states. This phenomenon resembles the experimental observation that progenitor cells pass through multiple intermediate states as they differentiate [5]. Moreover, these simulations also highlight that the transition of a triad from one state to another can take multiple possible routes. Similar results have been observed at the cellular level, where recent progress in de-differentiation [17], [19], [20] and trans-differentiation [77] has shown that natural developmental cellular state transitions may be only one of many possible routes between cell types [78].

Third, trajectories of the stochastic simulation showed a directional bias in gene expression state space, presumably due to mutual repression. For instance, when the system was in the X-ON/Y-ON/Z-OFF state, its trajectory was virtually limited to the X-Y plane, with little fluctuation in the Z direction (Figure 3C, middle and right panels). The result is that each state in these simulations was functionally “flat” and comprised of the expression from only two genes. Similarly, the one-ON states had a polar or single dimensional expression distribution.

Next, we subjected the system to a range of noise levels to gain a deeper understanding of the system's response and pattern of state transitioning. Noise strengths ranging from 0.4 to 1.2 were selected for analysis. Plotted in Figure 3D (red line) is the number of states traveled by the system, from the all ON state, within the simulated time versus the noise strength. The result shows a step function-like increase in the number of states with increasing noise strength. A similar pattern was observed when the simulation was initiated at the origin (blue line) rather than the all-ON state. One exception is that from this alternative starting point the system could not travel to the all-ON state and therefore had only six accessible states.

In both simulations, the sharp increase in the number of states traveled occurred between noise strengths of 0.8 and 1 (Figure 3D). This appears to be the critical threshold for overcoming the stability barrier between states and for complete noise-induced state transitioning. The existence of such a critical noise threshold suggests that natural systems may have the ability to operate in different regimes that either utilize or repress gene expression stochasticity to achieve distinct physiological goals.

### SSS Analysis

**Figure 4:**
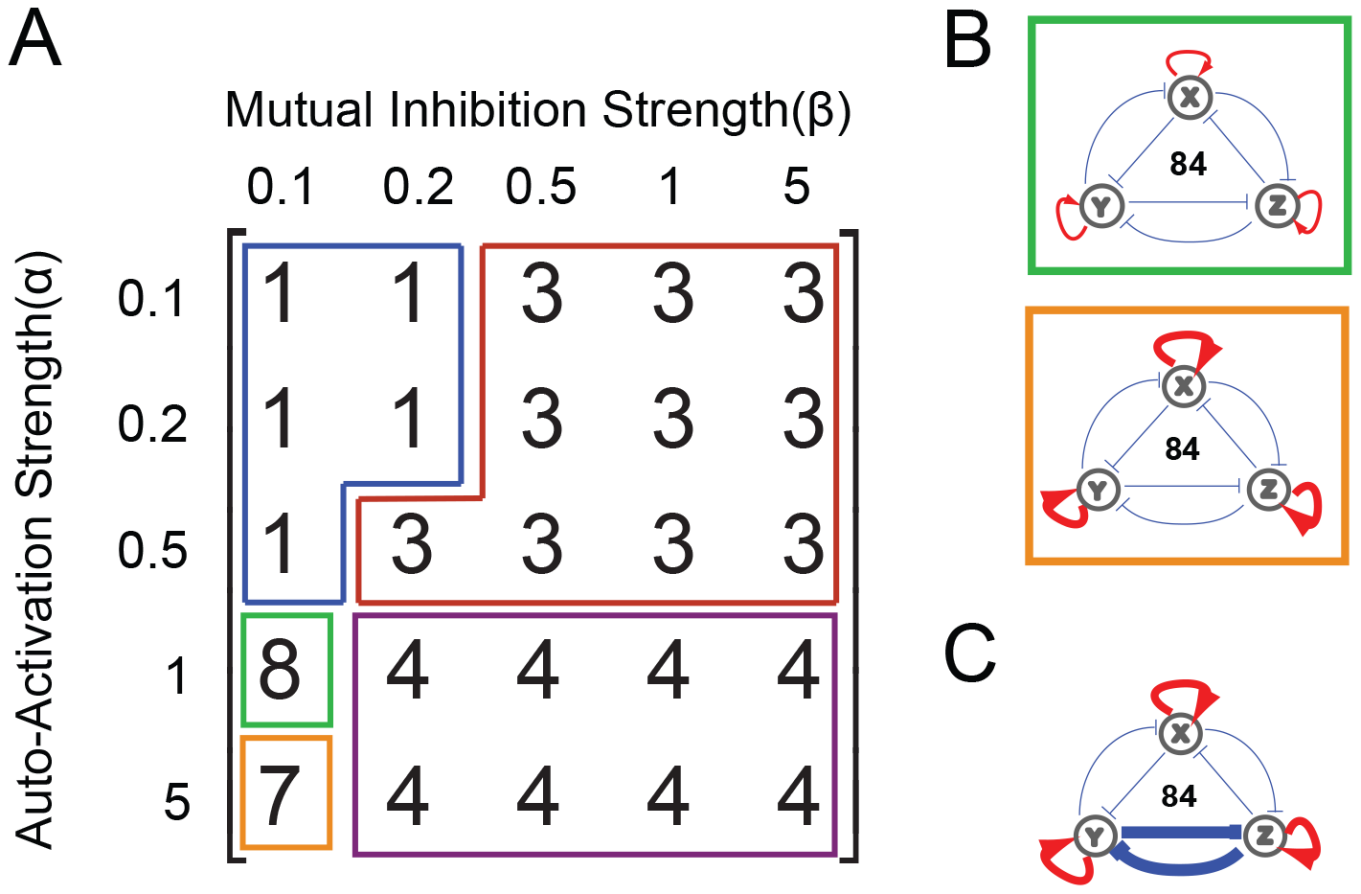
Stable Steady State Analysis of Network 84. (A) A matrix presenting the number of stable steady states generated by combinations of different auto-regulatory strengths (rows) and mutual-regulatory strengths (columns). This provides an overview of network stability at various points in the parameter regulation space; the green and orange regions are visualized as FCT diagrams independently in B. (B) The green-boxed diagram corresponds to the green-boxed parameter combination regime from (A), where genes have regulatory strengths α = 1 and β = 0.1. By contrast, the orange-boxed diagram corresponds to the orange-boxed parameter combination regime from (A), where nodes have regulatory strengths α = 5 and β = 0.1. This parameter combination regime allows the system to be stable in 7 of the 8 SSS, losing only the all-OFF state. (C) Many of the parameter combinations yielding multi-stable systems are not represented by the matrix, and are instead abstracted here. As an example, here we present a parameter combination regime that can support 6 SSS and auto-activation strengths are similar to those in the orange box (thick red arrows) but have one unrestrained pair of mutual inhibition that can exist at a very high strength (blue T-head repression lines).

To further explore Network 84's response to two or more simultaneously changing parameters, we conducted more thorough parameter scanning (see MATERIALS AND METHODS for details). First, two parameter combination regimes shown in Figure 4A and B suggest that, with strong auto-activation, Network 84 is ensured to have at least four states (bottom two rows). Under these conditions, weak mutual inhibition led to seven or eight states (orange and green boxes) while strong mutual inhibition led to only four: all two-ON-one-OFF combinations and all-ON (purple box). However, when both mutual inhibition and auto-activation are weak, Network 84 assumes only one state, that of all genes OFF (Figure 4A, blue box). When auto-activation is weak and mutual inhibition is strong the system is capable of the three two-ON-one-OFF states (Figure 4A, red box, locations of SSS not shown). Taken together, this again reinforces the importance of strong auto-activation in generating multistability and hence the potential to canalize cell differentiation. As a third parameter combination regime, we tested the effect of asymmetrical regulation on multistability (Figure 4C, see MATERIALS AND METHODS for details). Here we increased the mutual repression of a single pair of genes for the conditions represented by the green and orange boxes (Figure 4A, auto-regulatory strength between 1–5) and saw the number of SSS fall to six. Other similar simulations of asymmetrical regulation have been included in the supplementary information (Figure S3). These findings provide possible insight into why certain network topologies have been adopted by evolution for particular regulatory tasks. For example, Network 84 demonstrates a broad, multistable parameter space that can be tuned, through regulatory strengths, for the step-wise control of the maximal number of states. Such a mechanism could be envisioned to transit cellular identity from a pluripotent state to multi- or uni-potent state during differentiation.

### A Regulatory Network for Pluripotency

Experiments probing the structure and function of the regulatory network governing pluripotency suggest that the core pluripotency regulators Oct4, Sox2, and Nanog are organized into a regulatory topology of mutual activation that is shared with Network 1 selected by our screen, [21], [79]. This fully interconnected autoregulatory loop is believed to stabilize self-renewal, govern the bistable switch between ES cell self-renewal and differentiation, and underlie the ability of selected pluripotency regulators to ‘boot up’ the pluripotency transcriptional programming upon forced expression in somatic cells, thereby reprogramming them to a stem cell-like state [52]. Additional layers of regulatory complexity have been described in this network, including autorepression by Nanog [80], [81] and differential cell phenotypes in response to varying levels of these factors [82]–[84]. Moreover, Nanog acts as a homodimer [85], while Sox2 and Oct4 heterodimerize to regulate transcription [86], and attempts have been made to model the regulatory dynamics of these transcriptional complexes and their interaction with signaling factors [87]. Further adding to the complexity, Oct4 may also partner with other Sox factors to form alternative regulatory complexes that bind a distinct set of enhancers [88].

**Figure 5:**
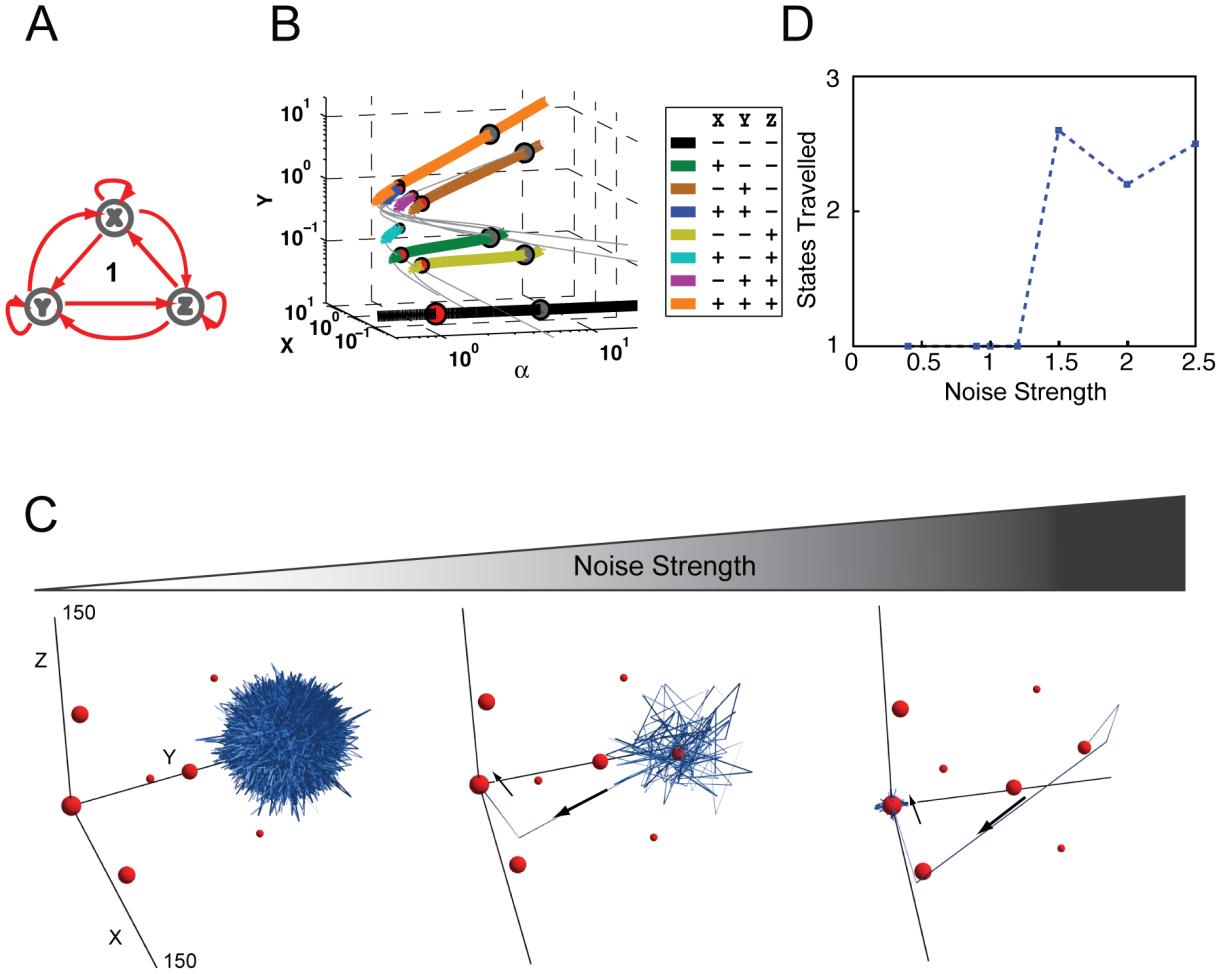
Bifurcation and Stochastic Analysis of Network 1. (A) The topology of Network 1 was also predicted to have high probability for multistability. Network 1 shares the topological architecture of the Oct4-Sox2-Nanog triad, and is believed to underlie stem cell differentiation and reprogramming. (B) As in Figure 3B, the X and Y protein concentrations of stable (colored) and unstable (gray) steady states at various self-activation strengths can be seen. A legend is shown as in Figure 3. (C) Simulations of noise-induced state transitioning in Network 1 under different levels of noise. Simulations were performed with auto-regulation equal to 1 and mutual regulation equal to 0.1, with noise levels of 0.5, 0.85, and 1 from left to right. The locations of the deterministically calculated states are indicated with red spheres, with their size being correlated to their spectral radius. The blue ribbons indicate the temporal trajectories of the simulations. The black arrows indicate initial direction of state transitions. Potential cloud shape for each SSS is illustrated in Figure S6B. (D) The number of states traveled in the stochastic simulations plotted versus noise strength.

Notably, protein levels of the core pluripotency regulator Nanog have been observed to fluctuate in cultured pluripotent stem cell populations [23], [24], [89]–[92], suggesting that this regulatory network is capable of adopting multiple states. To further explore possible roles of multistability in stem cell transcriptional regulation, we carried out detailed analysis on this network topology (Figure 5A). Our goal in doing so is not to capture every regulatory complexity present in the endogenous biological network, but rather to provide a theoretical framework for how this simplified network topology and fluctuations in network components might affect multistability. As with Network 84, we first carried out the bifurcation analysis for Network 1 (Figures 5B and S4). Considering the topology of Network 1 consists solely of positive regulation, one might expect that the network would primarily assume the all OFF “resting” or all ON “activated” states. This intuition is largely correct but not complete. While network 1 does tend toward the all-OFF state it is also capable of generating eight states when the strengths of auto-regulation and mutual regulation are 1 and 0.1, respectively (Figure 5B and C; 6A). This diagram (Figure 5B) shares some similarities with that of Network 84 (Figure 3B). For instance, both networks exhibit eight SSS when auto-activation strength is equal to 1, and fewer as auto-activation strength increases. Likewise, when auto-activation is reduced to be smaller than 1, both networks collapse to only support one SSS (the black line). Thus Network 1 also displays discrete emergence and collapse, or hysteresis, of SSS, allowing it to potentially serve as a foundation for irreversible cell fate decision-making. It is interesting that while the two-ON states (blue, cyan, and purple lines) collapse readily as α increases, the one-ON states (green, brown, and yellow lines) and the all-ON and all-OFF states (orange, black), are stable over a wide range of parameter values. It is also important to note that despite similarities in the state space locations of each of the eight SSS in Networks 1 and 84, there are considerable differences in their relative stabilities.

As with Network 84, we next incorporated different strengths of gene expression stochasticity, or noise, to simulate state transitioning (Figure 5C). The simulations were started from the all-ON state to mimic the all-ON pluripotent state of the Oct4-Sox2-Nanog triad. In the presence of small levels of noise, the system remained in the initial state throughout the duration of the simulation (Figure 5C, left panel). When the noise level was increased, the system was able to spontaneously transition to other states before settling in the all-OFF state, which represents the all-OFF terminal, or differentiated, state (Figure 5C, middle panel; Movie S2). When the noise level was increased further, the system transitioned out of the initial state much earlier toward the all-OFF state via an intermediate state (Figure 5C, right panel), confirming that the all-OFF state is indeed the terminal state and robust to different levels of noise.

It should be noted that simulation times were the same for each panel (Figure 5C). In the high noise level simulation (Figure 5C, right panel), the system reverted to the all-OFF state relatively quickly and exhibited small stochastic deviations that rarely exceeded the boundary of the sphere representing the state. This could perhaps be likened to the terminal state of the Oct4-Sox2-Nanog triad after cell differentiation, where expression of these genes is markedly depressed in gene expression profiles. Furthermore, for the modeling of cell fate decisions during development, the binary nature of these simulations appears to mimic the observed stable states of the Oct4-Sox2-Nanog triad, typically in either the all-ON or all-OFF state, with partial activation visible transiently during iPSC induction [17], [18], [93].

Much like the trajectories observed for Network 84, those for Network 1 exhibited the ability to transition between many states stochastically, with the potential of accessing five of the eight possible states. Although, as depicted, the residence time of states for Network 1 was largely restricted to either all-ON or all-OFF (Figures 3C, 5C). Network 1 simulations could not access the three two-ON-one-OFF states unless initiated there, presumably because of the states’ low stabilities. Moreover, unlike Network 84, all the simulation trajectories for Network 1 ultimately reached and remained at the all-OFF state, a result of Network 1's topology and the superior stability of the all-OFF state. This also led to a lower average number of states traveled by Network 1 at higher noise levels (Figure 5D). A higher noise threshold for state switching of this sort could serve as added protection against aberrant regulation of pluripotency. From a modeling perspective, these results offer a useful means of rapidly predicting the dynamical stochastic trajectory of a network based on state stabilities, without having to run entire stochastic simulations.

**Figure 6:**
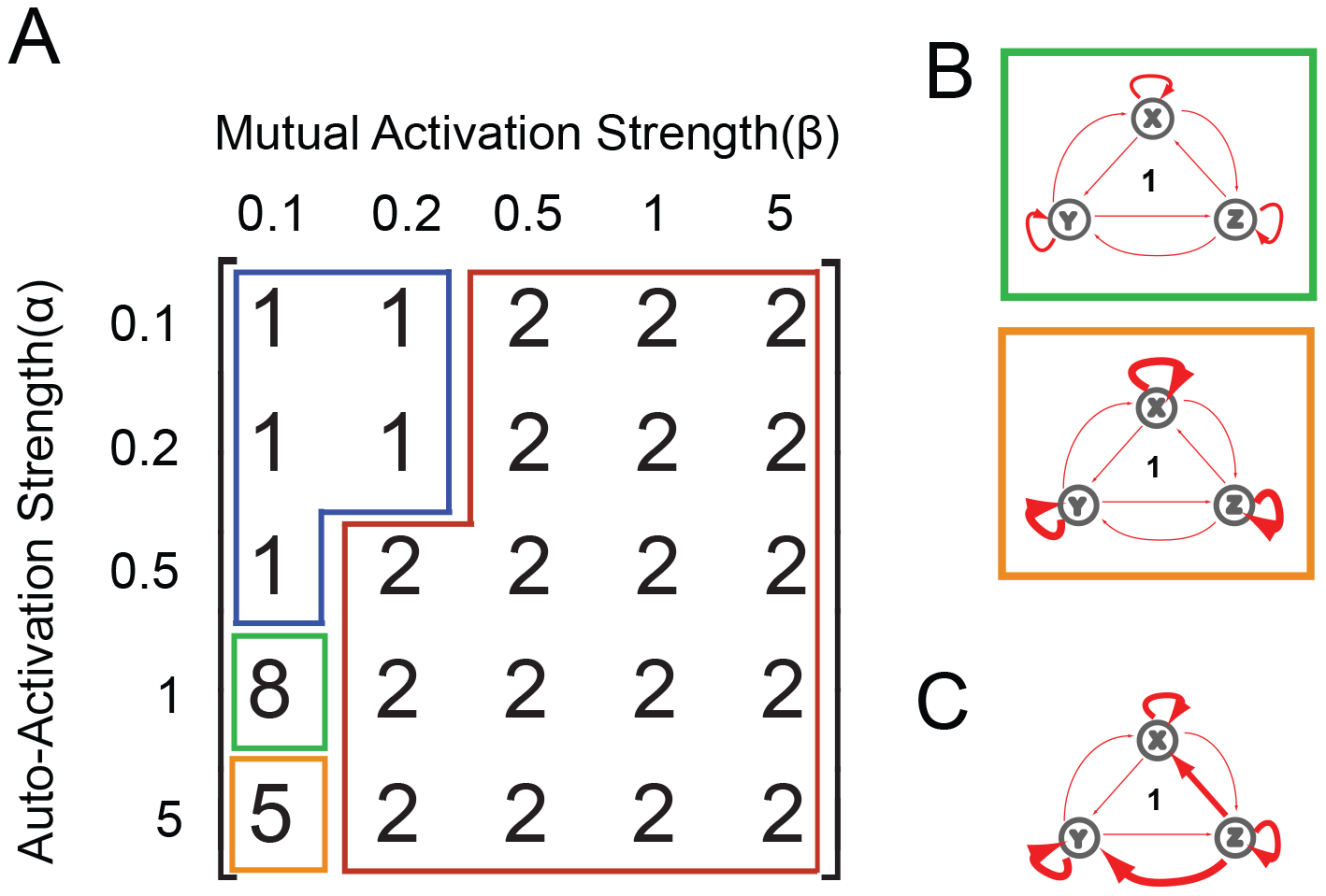
Stable Steady State Analysis of Network 1. (A) A matrix defined by examining the number of SSS present at points where all auto-regulatory strengths (row) are equal and mutual-regulatory strengths (column) are also equal. This provides an overview of network stability at various points in the parameter regulation space; the green and brown regions are visualized as FCT diagrams independently in B. (B) The green-boxed diagram corresponds to the green-boxed parameter combination regime from (A), where genes have regulatory strengths α = 1 and β = 0.1. The orange-boxed diagram corresponds to the orange-boxed parameter combination regime from (A), where nodes have regulatory strengths α = 5 and β = 0.1. This parameter combination regime allows the system stability in 5 of the 8 Stable Steady States, losing only the two-ON states. (C) As in Figure 4C, there exist parameter combinations not captured by the matrix representation, where increased strength of any one of the five regulations (represented by thick arrows) will enable 5 SSS.

As with Network 84, we tested the sensitivity of Network 1 to different combinations of regulation strengths (Figure 6). Again, weak mutual activation and strong auto-activation gave rise to the maximal number of states (Figure 6A, green and orange boxes). Interestingly, however, the remainder of the regulation parameter space was much simpler than that of Network 84, yielding only one or two states (Figure 5A, blue and red boxes). Again, independent manipulation of mutual regulatory interactions can also lead to parameter combinations that enable multistability. A thorough parameter scan for such multistability found a third interesting configuration, one where a gene (Z) propagates signal to downstream genes (X and Y), which also have strong auto-activation (Figure 6C). This pattern has one defining characteristic in that it maintains multi-stability as long as there is only one master while the promoted genes have weak interactions with each other. Such multi-stability is lost when the system adopts a two-driver gene topology. Therefore Network 1 may be considered a largely bimodal topology that retains the potential for greater state diversity, but only within a narrow parameter window (Figure 5B and 6A). This characterization of Network 1 topology is supported by other studies. Specifically, while claims of monoallelic expression of Nanog [89] have been contested [24], [76], a common finding across the literature is heterogenous expression of endogenous Nanog protein in cultured ES cells by immunostaining [23], [24], [75], [76], [89], [90]. These findings suggest bistable expression of Nanog, and support the idea that the Oct4-Sox2-Nanog network may exist in at least four stable states (all high; Oct4 and Sox2 high but Nanog low, as occurs in low Nanog ESCs; high Sox2 but low Nanog and Oct4 as occurs in neural progenitor cells [94]; or all low/off in the differentiated state).

Analysis of the regulatory parameter space for networks 1 and 84 raises an important consideration that may be broadly applicable. Could progressive lineage determination, or restriction of pluripotency, be correlated with the strength of mutual regulation? For both of the networks analyzed, the highest potential for multistability (eight states) was found in a parameter space with weak mutual regulation (Figures 4A and 6A). As the strength of mutual regulation increases from this point, more restricted potential fates predominate, much as would be expected as cell lines differentiate. The influence of greater mutual regulation on the potential for multistability would be interesting to explore, and perhaps is well-suited to modeling in the hematopoietic system where hematopoietic stem cells (HSCs) or multi-potent progenitors could be compared with bi-potent progenitors.

### *In Silico* Induced Pluripotency

**Figure 7:**
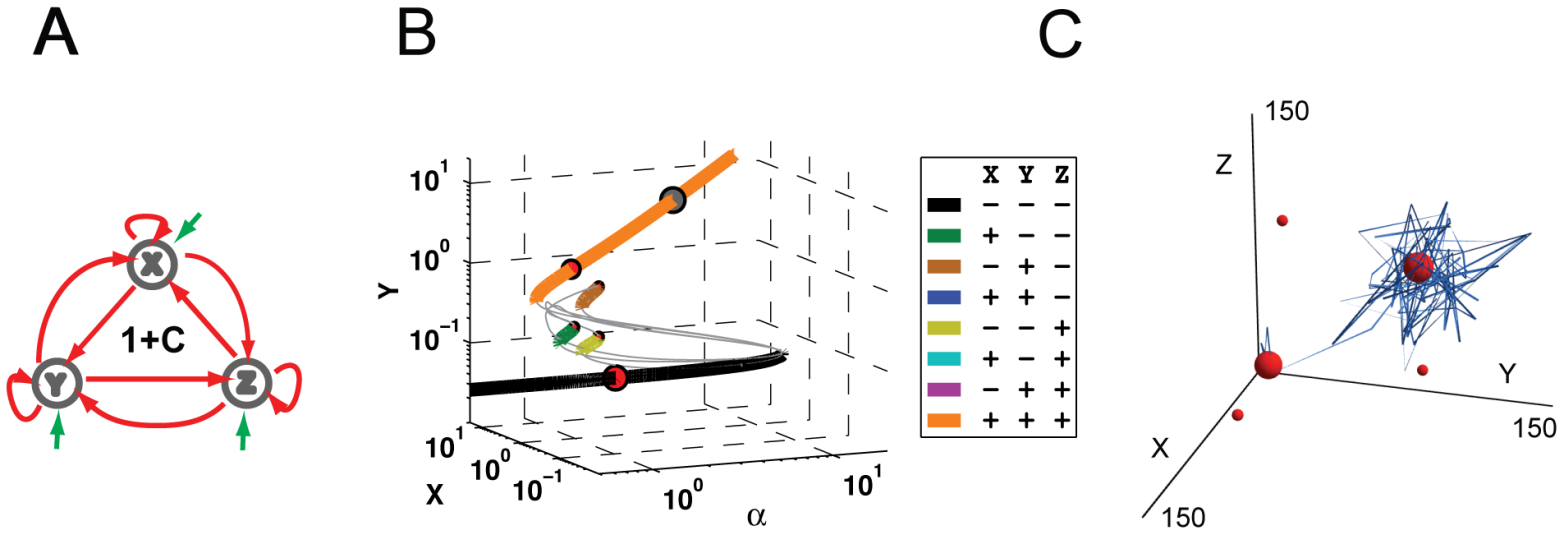
Bifurcation and Stochastic Analysis of Network 1 with constitutive basal expression. (A) The topology of Network 1 is used to mimic iPSC experiments with added constitutive expression of all three genes. (B) As in Figure 5B, the X and Y protein concentrations of stable and unstable steady states are plotted against self-activation strengths. With the addition of the constitutive expression we can see a further decrease in stabilities, specifically two-ON states are never stable. Additionally one-ON states lose stability very rapidly, approximately at α = 1, and the all-OFF state destabilizes after α = 9 leaving only the all-ON state. This can also be seen in the sizes of the spectral radii, deterministically these steady states exist but in practice they have very little influence. (C) Simulations of Network 1 modeled in the presence of an additional constitutive overexpression term consistently show a transition from an all-OFF state to an all-ON state after a period of latency.

Reprogramming mature cell types into iPSCs is achieved by the overexpression of a number of defined transcription factors (typically including Oct4, Sox2, c-Myc, and Klf-4) that regulate the necessary pluripotency phenotypes as well as each other's expression [18]–[20]. Recently, it has been shown that overexpression of only Oct4, Sox2, and Nanog results in similar rates of iPSC generation from human amnion cells [95], and, importantly, the Oct4-Sox2-Nanog triad is highly expressed and appears to be the core regulatory circuit for pluripotency in stem cells [96]. To construct a simplified *in silico* version of the iPSC experiment involving the Oct4-Sox2-Nanog triad, we added a constitutive expression term to each gene of Network 1 (Figure 7A, see MATERIALS AND METHODS).

To begin, we performed bifurcation analysis on the network 1 topology with an added term for constitutive expression (Figures 7B and S5). The addition of a basal level of expression to Network 1 resulted in at most five possible states, two of which (the orange all-ON and black all-OFF lines) possessed considerably higher stabilities than the others (Figure 7B). All one-ON-two-OFF SSS (green, brown, and yellow lines) are only stable for a narrow range of α. Even when they are stable, their stabilities are insignificant. Looking closely we can see that between the red, green, and yellow lines there are 3 unstable paths without a stable component. These lines would be the locations of two-ON-one-OFF states if they achieved stability. More importantly, as auto-activation strengthens, even the all-OFF state's stability becomes insignificant when compared with the all-ON state (Figure 7B gray circles).

A comparison of bifurcation diagrams of Network 1 with and without the constitutive expression term (Figure 5B, 7B) highlights some striking and important differences. Firstly, as observed experimentally, by adding the constitutive basal expression term to all three genes, there is increase in the stability of the all-ON state. Moreover, our simulation predicts a reduction in the number of possible SSS from eight to five, as well as a significant reduction in the stability of the non all-ON or all-OFF states. While these findings must be considered within the limits of our study, they suggest constitutive expression could help to canalize the transition from all-OFF to all-ON by simplifying the triad's regulatory space, and thus provide a foundation for induced pluripotency.

To further examine modified Network 1, the effects of gene expression noise were investigated through stochastic simulation from the all-OFF state with a moderate level of noise (see MATERIALS AND METHODS). Consistent with experimental evidence [34], at the start of the simulation, the system hovered near the origin for a period of time and then began to transition toward the all-ON state. Once again, by adding a basal overexpression term to the genes, we found that the stability of the all-ON state was increased to be greater than that of the all-OFF state (Figure 7C; Movie S3). As a result, the system was attracted to the all-ON “pluripotent” state in a way that was not observed with the unmodified Network 1 (Figure 5C).

These findings reflect observations from the experimental practice of induced pluripotency in which the reprogramming factors are strongly overexpressed, and may shed light on the regulatory mechanisms behind induced pluripotency. For instance, the overexpression of defined transcription factors in differentiated cell types may alter the regulatory parameters of the FCT into ones that prefer the all-ON “pluripotent” state. Affected cells would then gradually transition from their original state to the preferred all-ON state. Further, as is often done experimentally, we removed the constitutive expression term from our simulation and found the system to return to an unmodified Network 1 behavior. Much like iPSCs, the network was able to re-differentiate to the all-OFF state or to remain in the all-ON state, under conditions of low noise or high auto-activation respectively.

**Figure 8:**
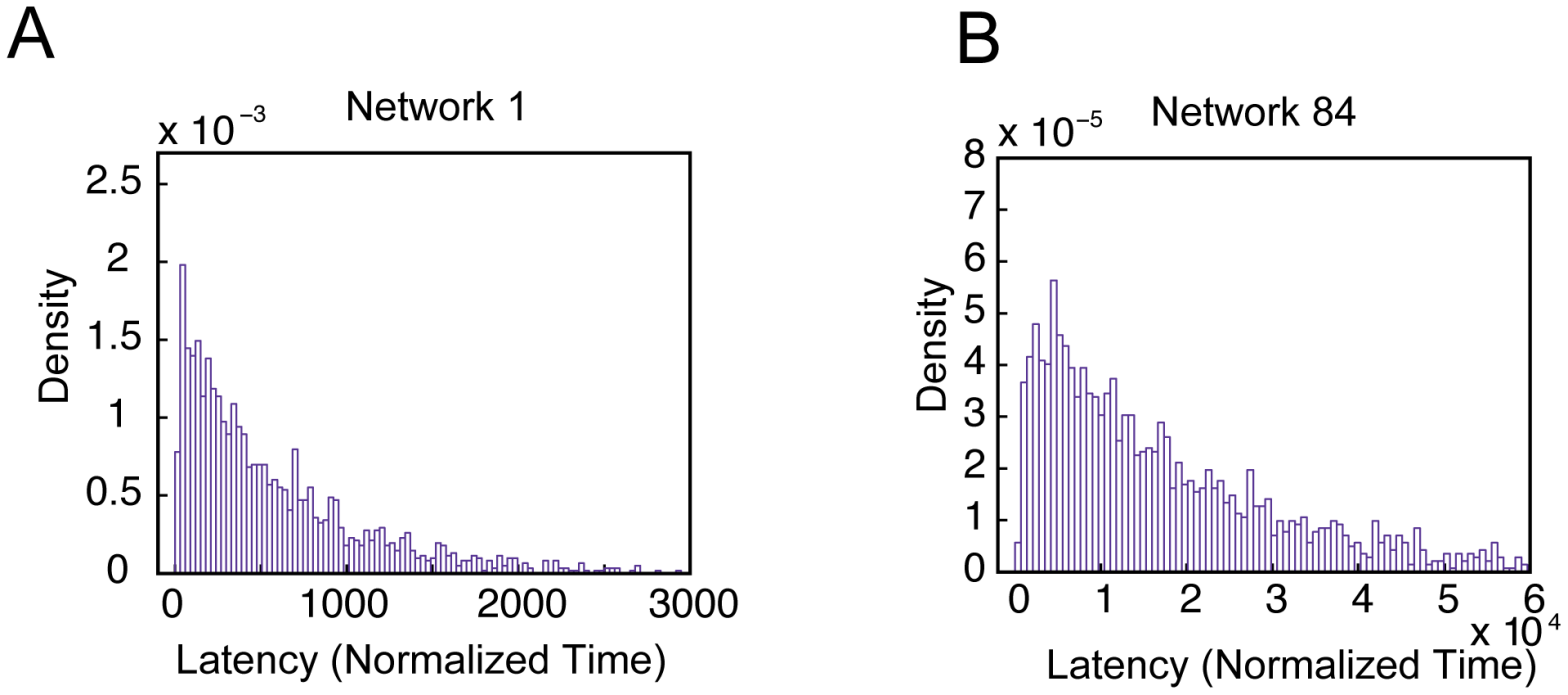
Stochastic simulations capture latency distributions in induced pluripotency. (A) Distribution of simulated latency for the modified Network 1 model illustrates a skewed bell shape. The histogram was generated from 2000 simulations. (B) Similar results generated from 2000 simulations with Network 84.

To further test the utility of our model as an abstraction for the transcriptional regulation of stem cells and stem cell reprogramming, we next used our model to predict the temporal dynamics of stochastic reprogramming. We first investigated the time required for the system to transition from the all-OFF to the all-ON state, which is termed the first passage time (FPT) [97]. An FPT distribution of induced pluripotency was generated by running 2000 simulations with our modified Network 1, resulting in a characteristic asymmetrical bell shape distribution with a long tail and a peak at approximately 50 normalized time units (Figure 8A). To ensure the generality of this observation, an FPT distribution for the transition from one state to another in Network 84 was similarly generated and found to yield the same pattern (Figure 8B). Such a skewed bell shape distribution is consistent with published experimental data [34].

## Discussion

Over fifty years have passed since the connection between development and multistability was first proposed [3]. However, despite efforts in the last decade to reverse engineer transcription regulatory networks, the molecular basis for multistability is still not well understood. Likewise, multistable systems with more than two states have not yet been constructed *de novo*. We address this challenge with a high-throughput, dynamical systems search algorithm, powered by parallel computing, that identifies gene network topologies enriched in their capacity to realize multistability.

Focusing our screen on FCTs, our algorithm identified complete auto-activation as an important topological feature for high-dimensional multistability, along with a variety of combinations of mutual regulation. Our analyses shed light on existing ideas and generate new testable hypotheses for some of the regulatory mechanisms underlying cell fate determination. Many of the seemingly disparate experimental observations including random reprogramming latency, the existence of intermediate states during differentiation and reprogramming, population heterogeneity, and alternate routes of cellular state transitioning [5], [27], [34], [96], [98], can potentially be connected and addressed by our dynamical analyses of FCTs.

The central importance of complete auto-activation in multistable triads suggests that nodes for such regulatory networks could be identified in exogenous overexpression screens for known key regulators. In such a screen, the components of multistable FCTs would self-identify, by maintaining their own expression after the withdrawal of candidate transcription factor overexpression. Transcription factors identified this way could represent “pioneers”, those capable of activating regulatory partners or overcoming transcriptional inaccessibility, i.e., silenced loci, such as Oct4.

The identification of regulatory partners (mutual regulation) could also be characterized by the analysis of promoter/enhancer regions upstream of identified auto-activation transcription factors. Such a “guilt by association” method may also help to infer the function of the triad as a whole, or lesser-known regulatory partners. Thus, using established developmental transcription factors as seeds, the Waddington landscapes of whole cell lineages could perhaps be mapped by using auto-activation as a cipher.

The notion that specific topologies may serve particular regulatory roles better than others is supported by the identification of an additional Network 1 regulatory topology in a stem cell population. The transcription factors Gata2, Fli1 and Scl/Tal1 form a regulatory triad that operates through mutual and auto-activation to promote hematopoiesis in the mouse embryo [99]. As discussed in the context of the Oct4-Sox2-Nanog triad, the Network 1 topology has a tendency to operate in a bimodal and terminal manner, meaning that once switched to the all-ON state the triad has a tendency to be locked into that state. Such a regulatory system would impart a sort of cell “memory”, perhaps allowing newly formed HSCs to travel from the aorta-gonad-mesonephros (AGM) or primordial niche to the fetal liver and bone marrow niche without losing their multipotent state [99], [100].

Importantly, regulatory triads do not exist in isolation and are not static entities. Rather, inputs from the environment and other signaling pathways can influence the state or composition of endogenous triads through the perturbation of regulatory relationships. The consequence of such influence was evident in our bifurcation analysis of Networks 84, 1, and modified 1, where small changes to the strengths of the network regulation created or eliminated SSS. Thus, under the influence of external signaling (e.g., post-translational modification), subcellular localization of co-factors or regulatory ligands [101]–[103], the same network topology may alternate between different regulatory state configurations. Such state switching could be an effective regulatory strategy for responding to changing environmental and developmental cues.

While gene expression stochasticity is an accepted factor of cell fate determination [61], its role in complex processes such as differentiation remains elusive. We subjected our chosen multistable networks (e.g., Network 84) to stochastic simulations and found that moderate levels of gene expression noise were sufficient to disrupt the balance of protein abundances required for maintaining all-ON and all-OFF states, even with high protein levels. In other words, our simulations suggest that noise is critical to a biological system's ability to transition from meta-stable states to differentiated states. Moreover, noise-induced transitioning showed multiple possible routes from one state to another, with the trajectories traversing step-wise through adjacent states (Figures 3C, 5C). This is possibly the *in silico* manifestation of experimental observations showing progenitor cells passing through alternate intermediate states on the way to a differentiated state [5]. Additionally, by testing a range of noise levels, we found that the number of states traveled by these multistable systems increased sharply at specific noise levels (Figures 3C, 5C). Perhaps cellular systems straddle such a threshold, whereby gene expression noise can either be suppressed or amplified to realize very different physiological outcomes. The advent of single-cell gene expression profiling methods [104] affords new windows on cellular heterogeneity, and theoretical frameworks such as we present here may provide valuable insights when informed by the experimental data these technologies will provide.

Like signaling cascades in their simplest form, the regulatory relationships between the genes of FCTs define their meta-properties. In a manner reminiscent of the low-pass filtering capacity of cascades, our stochastic simulations detected network-specific variation in tolerance for noise. Specifically, in the analyses of Networks 1 and 84, significantly different noise thresholds for multistability were measured—0.8 and 1.4, respectively (Figures 3 and 5). This observation suggests that, along with other measures, networks may be tuned for unique noise-switching thresholds. For example, the Gata2-Fli1-Sci/Tal1 triad (Network 1) would be predicted to remain in the all-ON state as long as noise levels are maintained low, but to transition quickly to an all-OFF state under high noise. This could be tested experimentally using tunable synthetic noise controllers [94] or generators [105] upstream of these HSC regulators to apply large fluctuations to the system.

As such, differentiation or pluripotency may provide readily accessible experimental models. One possible method to induce noise could be the expression of splice isoforms [106], [107]. Focusing on the endogenous triads discussed, state change dynamics could be evaluated under the perturbations of either single or multiple splice isoforms. Thus, while controlling for total protein expression within a particular triad, one could evaluate whether “noise”, derived from the concurrent expression of multiple isoforms, influences the product or rate of the induced state transition. If so, an interesting outcome may be that individual differentiation pathways could be primed for state change by expressing tailored suites of splice-isoforms or in the case of iPSC reprogramming, perhaps accelerate the rate of state switching.

Finally, our work offers new directions for synthetic biology [11], [12], [108]–[112]. As a tool, the application of synthetic biology to disease-related or developmental triads may allow for true reverse engineering of *in vivo* processes. For example, the regulatory dynamics of Network 1 (Gata2-Fli1-Scl/Tal1 and Oct4-Sox2-Nanog) or Network 84 (RUNX2, SOX9 and PPAR-γ) topologies could be systematically examined for response to perturbation or noise by constructing such FCTs in an orthogonal environment (e.g., bacteria). Similarly, the effects of stochasticity on the regulation of pluripotency could be studied directly in mammalian systems using tunable synthetic noise generators. Such a system could vary the gene expression of triad components, while keeping their mean expression level constant, to evaluate the influence of noise on network state change [57], [113]. Our work here provides a starting point for the design of next-generation synthetic circuits that could exploit multistability and its physiological consequences.

## Materials and Methods

### Enumerating and Eliminating Redundant Networks

Three-node networks can have 3^9^ = 19683 configurations, each with nine edges that consist of positive, negative or null regulations between the nodes. Our focus on FCTs resulted in a total of 2^9^ = 512 possible configurations because each edge can only represent positive or negative regulation. Through permutation analysis many of these 512 networks were found to be identical to each other. Identical networks were eliminated, leaving us with 104 unique networks (see SI).

### ODE Modeling and High-Throughput Screening

A three-node gene regulatory network can be described by a three-dimensional ODE:
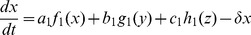
(1)

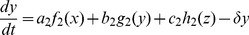
(2)

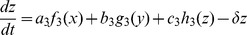
(3)


In this formulation each variable (*x*,*y*,*z*) represents the protein abundance of one gene product (Equations 1–3). Here only one generic equation is used to describe the gene expression regulation including transcription, translation, and other post-transcriptional and post-translational modifications. Each equation captures the core nonlinear dynamics of gene expression regulation and omits details of the fine-tuning of it. The parameters (*a_1–3_, b_1–3_, c_1–3_*) represent the strength of mutual or auto-regulation, be it activation or inhibition. Each function (*f*, *g* or *h*) has the form F^n^ / (k^n^ + F^n^) when representing activation, and the form k^n^/(k^n^+F^n^) when representing inhibition, where *F* can be either *x*, *y*, or *z*. The activation and repression of each gene by other factors are modeled additively to account for reported mechanistic independence of transcription regulations [16], [40], [46]. For example, Network 84 shown in Figure 3A can be described by the ODE:
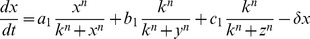
(4)

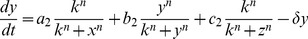
(5)

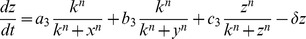
(6)


In the models values of *n = 4* and *k = 0.5* were assumed for all genes (Equations 4–6). While it is known that transcription factors critical in stem cell programming often form homodimers [85], [114] or heterodimers [115], *n* is set to be equal 4 because the nonlinearity quantified by *n* is often affected by many factors in addition to protein multimerization [11]. A hill coefficient of 4 has been used previously to demonstrate generic behavior of stem cell differentiation [16]. Recently, hill coefficients up to 9 have also been used [40] to construct models with experimentally verified predictions. It is also found in our study that hill coefficient values affect the region of multistability but not general conclusions. While it did not affect the results of steady-state calculations, δ was set to be 1; this assumption can be adjusted in light of future available experimental results. For mutual regulation strength, the parameters (*b*_1_, *c*_1_, *a*_2_, *c*_2_, *a*_3_, *b*_3_) were set to [0.1, 0.2, 0.5, 1 or 5]; for auto-regulation strength, the parameters (*a*_1_, *b*_2_, *c*_3_) were set to 0.1, 0.5 or 1. These parameter possibilities lead to a total of 5^6^*3^3^ = 421,875 parameter sets to test.

We numerically solved for the root of the right-hand side of each ODE set with over 1000 different initial guesses uniformly distributed over the entire state space. Measuring the probability of multistability for each FCT was accomplished by analyzing all 421,875 parameter sets. A parallel algorithm was developed to distribute this task as an ensemble of independent computations (See the SI).

### Analysis of the Parameter Space

In addition to the tabular data (Figure 4A and 6A) that is useful for quick visualizations and analysis of stabilities, it is desirable to recognize key patterns of parameter combinations (PCs) yielding multistability. Therefore networks chosen (Network 84 and 1) for in-depth analysis had their results recomputed with a wider sample of parameter spaces. Specifically the PCs were expanded to five levels for each edge, [0.1 0.5 1 2 6], yielding 5∧9 = 1,953,125 possible PCs. The found clusters can then be generalized to create the FCT diagrams in Figures 4C and 6C.

### Bifurcation Analysis

Bifurcation diagrams were generated using MatCont [116], a continuation toolbox for MATLAB. Bifurcation analysis was performed on the auto-activation strength; mutual-regulation strengths were set at .1. Spectral radii were gathered at specific intervals to compare the stability of the stable steady states. Stable and unstable steady state information was graphed to generate Figures 3B, 5B, and 7B, for more information please see SI.

### Stochastic Simulations

The ODE model can be converted to chemical Langevin equations to introduce stochasticity while approximating the Gillespie algorithm [68], [117]. All parameters were scaled up so that copy numbers of gene products are in biologically reasonable quantities and can reach over one hundred. This was done by multiplying the coefficients in *a*, *b*, *c* and *k* by 100 with other parameters unchanged. This linear shift does not alter the bifurcation dynamics or the relative locations of SSS. At each iteration, all chemical species are updated using the equation: 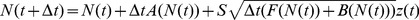
(7)


In this equation, *N*(*t*) represents the abundance of chemical species, *A*(*t*) is the right-hand side the ODE, and *F* and *B* are the forward and backward reaction terms in each equation, respectively, which were extracted from corresponding ODEs. *z*(*t*) are the standard Normal variables, *S* is the stoichiometric matrix of the biochemical reactions (refer to [68] for additional details). In our modified algorithm, we added one parameter, α, to Equation (13) to represent the tunable noise strength:
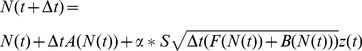
(8)


Here α was the noise strength used in Figures 3C and 5C. Therefore, the results were comparable to pure discrete simulations when α equals 1. Modulation of the noise strength(α) was used to facilitate investigations of the system's dynamics in response to different noise levels. The multiplicative noise term is chosen for simplicity without loss of generality.

### Statistical Tests

All statistical tests were performed using Matlab. The parameters for each distribution were estimated using the Matlab command *mle*. Chi-square (*X* ^2^) statistics for each distribution were computed with the *chi2gof* command.

## Supporting Information

Figure S1 An example of the connection matrices and topological permutations used to identify unique FCTs. In the two FCT connection matrices illustrated, each row represents the source of regulation and each column the recipient of regulation, with symbols representing the activation or repression. As indicated by the arrows, these two matrices are permutations of each other after switching the Y and Z labels. Complete permutation analysis involved both row and column switching.
(PDF)

Figure S2 Bifurcation diagram in Figure 3B shown with different viewing angles and visual assistance. (A) The bifurcation diagram of Figure3B is illustrated again to provide a frame of reference; the figure legend is consistent among all subfigures. (B) Bifurcation diagram is shown from the same angle as in A with planes drawn at α = 1 and α = 5 where the red and gray points lie precisely on the plane. (C) Bifurcation diagram drawn with a different angle to emphasize differences in y-locations of each SSS. Three planes drawn at y = 0.1, 1, and 5. Two gray points and two red points lie on the bottom plane, four red points lie on the middle plane, the SSS from the orange and blue lines overlap due to our projection, and four gray points lie on the top plane, again two points are overlapping, and one is out of the bounds of the graph. (D) Bifurcation diagram drawn with another different angle to emphasize differences in x-locations of each SSS. Three planes drawn at x = 0.1, 1, and 5. Four gray points lie on the left plane, with two overlapping, and one out of the bounds, four red points lie on the middle plane, with two overlapping, and two gray and two red points lie on the right plane.
(PDF)

Figure S3 Perturbations to network regulatory strengths affect the probability distribution of network states. Regulatory strengths for Network 84 were perturbed and the corresponding state probability distributions were generated using results of stochastic simulation. (A)–(C) Parameter strengths are identical to the green box in Figure 4, with simulation state plotted as point collections in B, and presented as a pie chart in C. (D)–(F) Random small perturbations to the regulatory strengths and corresponding state probability distributions. (G)–(I) Doubling in the strength of Y's inhibition of Z and corresponding state probability distributions. The magnitudes of perturbations are labeled on each network edge and are also represented by edge thickness. Axis labels of E and H are the same as those of B. Simulations for these distributions were calculated in the presence of a noise strength equal to 1, and each perturbation was simulated ten times. The point collections are color-coded based on their closest stable steady state, and their probabilities are summarized in panels C, F and I, respectively.
(PDF)

Figure S4 Bifurcation diagram in Figure 5B shown with different viewing angles and visual assistance. (A) The bifurcation diagram of Figure5B is illustrated again to provide a frame of reference; the figure legend is consistent among all subfigures. The position of the all-OFF states have been shifted due to the logarithmic graphing, and that the all-OFF state remains at 0 concentration of x,y, and z regardless of alpha. (B) Bifurcation diagram is shown from the same angle as in A with planes drawn at α = 1 and α = 5 where the red and gray points lie precisely on the plane. (C) Bifurcation diagram drawn with a different angle to emphasize differences in y-locations of each SSS. Three planes drawn at y = 0.1, 1, and 5. Two gray points and three red points lie on the bottom plane, four red points lie on the middle plane, the SSS from the orange and blue lines overlap due to our projection, and two gray points lie on the top plane. (D) Bifurcation diagram drawn with another different angle to emphasize differences in x-locations of each SSS. Three planes drawn at x = 0.1, 1, and 5. Two gray points lie on the left plane, four red points lie on the middle plane, with two overlapping, and two gray and two red points lie on the right plane.
(PDF)

Figure S5 Bifurcation diagram in Figure 7B shown with different viewing angles and visual assistance. (A) The bifurcation diagram of Figure7B is illustrated again to provide a frame of reference; the figure legend is consistent among all subfigures. (B) Bifurcation diagram is shown from the same angle as in A with planes drawn at α = 1 and α = 5 where the red and gray points lie precisely on the plane. (C) Bifurcation diagram drawn with a different angle to emphasize differences in y-locations of each SSS. Three planes drawn at y = 0.1, 1, and 5. One gray point and one red point lie on the bottom plane, two red points exist between the lower and middle planes (approximately 0.3), two red points lie on the middle plane, and one gray point lies on the top plane. (D) Bifurcation diagram drawn with another different angle to emphasize differences in x-locations of each SSS. Three planes drawn at x = 0.1, 1, and 5. One gray point lies on the left plane, two red points lie on the middle plane, two red points exist between the middle and right planes and one gray point and one red point lie on the right plane.
(PDF)

Figure S6 Visual representation of constrained steady state sub-space under noise perturbations. (A) Trajectory clouds displayed in Figures 3C are approximated by 3D ellipsoids. The radius in each dimension is correlated with the inverse of the strength of the eigenvalue corresponding to a particular eigenvector. i.e. if the eigenvalue for an eigenvector is only weakly negative, then the cloud will be large in the direction of that eigenvector. Here the all-OFF cloud is scaled down in size manually so that it fits on the graph. The all-OFF and all-ON states are both disk-shaped, with their primary eigenvalue being significantly stronger and facing [1,1,1]. (B) Similarly, 3D ellipsoids were used to visualize possible cloud shape shown in Figure 5C. Different scaling parameters are used due to 1-ON and 2-ON states having a large difference in eigenvalue strength. Even with the modified parameters the 2-ON states are barely visible in the graph. This is consistent with stochastic simulation results shown in Figure 5C, where the trajectories barely spend any time in any of the 2-ON states.
(PDF)

Movie S1 A movie showing the simulated temporal evolution of chemical species in Figure 3. The temporal evolution of transcription factors is shown as an elongating blue ribbon. All conditions are the same as in the right panel of Figure 3C. Only the first 5000 time points were animated.
(MOV)

Movie S2 A movie showing the simulated temporal evolution of chemical species in Figure 5. The temporal evolution of transcription factors is shown as an elongating blue ribbon. All conditions are the same as in right panel of Figure 5C. Only the first 5000 time points are animated.
(MOV)

Movie S3 A movie showing the simulated temporal evolution of chemical species in Figure 7. The temporal evolution of transcription factors is shown as an elongating blue ribbon. All conditions are the same as in Figure 6A. Noise strength is 1.5. Only the first 200 time points are animated because the simulation equilibrated rapidly.
(MOV)

Text S1 Supplementary texts of Materials and Methods.
(DOCX)
