# Supplementary figures and images for: Gene Networks of Fully Connected Triads with Complete Auto-Activation Enable Multistability and Stepwise Stochastic Transitions
Source: PLoS One. 2014 Jul 24;9(7):e102873. doi: 10.1371/journal.pone.0102873 (PMC4109943; doi:10.1371/journal.pone.0102873)

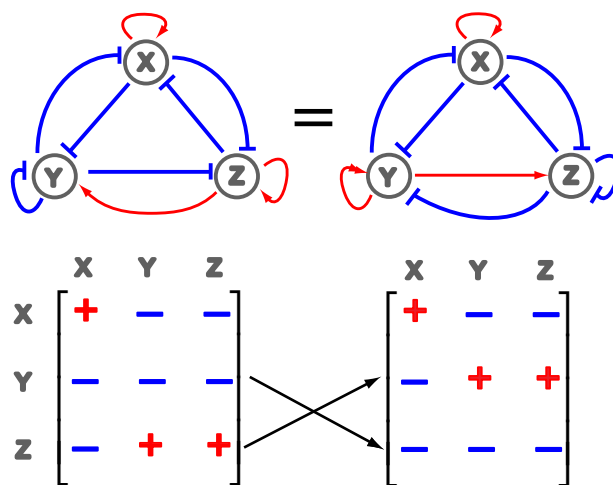

Figure S1  
Faucon *et al.*

Supplement: Figure S1 — An example of the connection matrices and topological permutations used to identify unique FCTs. In the two FCT connection matrices illustrated, each row represents the source of regulation and each column the recipient of regulation, with symbols representing the activation or repression. As indicated by the arrows, these two matrices are permutations of each other after switching the Y and Z labels. Complete permutation analysis involved both row and column switching. (PDF) [file pone.0102873.s001.pdf]

A

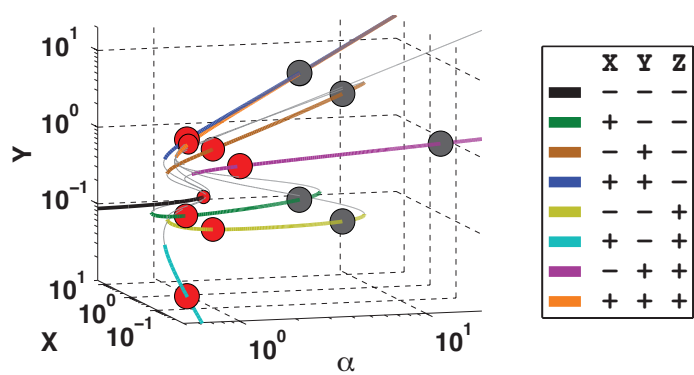

B

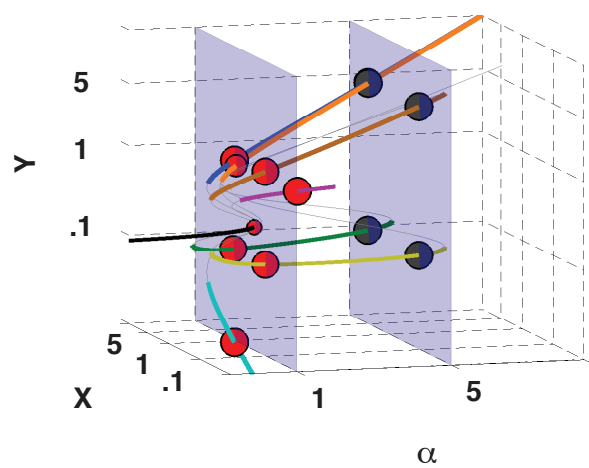

C

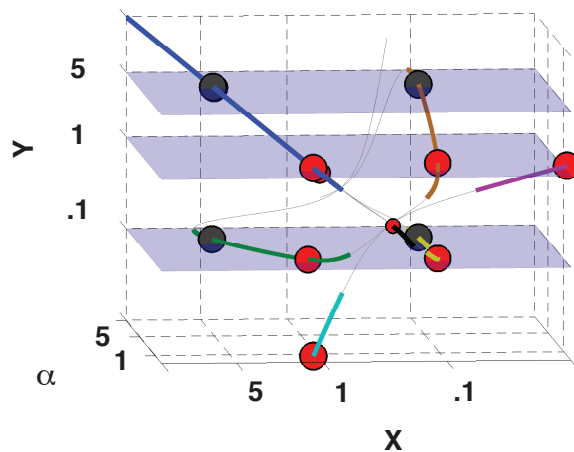

D

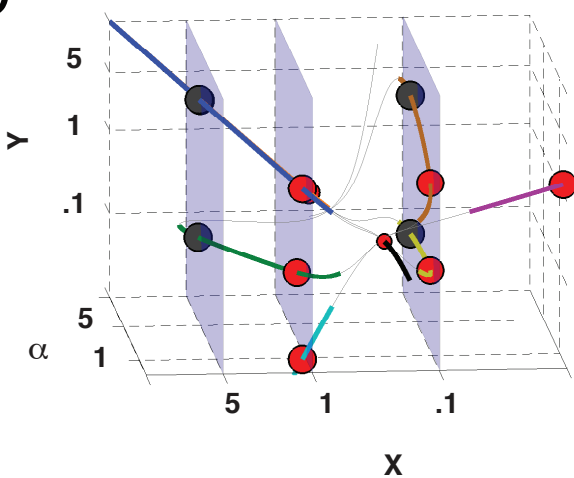

Figure S2  
Faucon *et al.*

Supplement: Figure S2 — Bifurcation diagram in Figure 3B shown with different viewing angles and visual assistance. (A) The bifurcation diagram of Figure3B is illustrated again to provide a frame of reference; the figure legend is consistent among all subfigures. (B) Bifurcation diagram is shown from the same angle as in A with planes drawn at α = 1 and α = 5 where the red and gray points lie precisely on the plane. (C) Bifurcation diagram drawn with a different angle to emphasize differences in y-locations of each SSS. Three planes drawn at y = 0.1, 1, and 5. Two gray points and two red points lie on the bottom plane, four red points lie on the middle plane, the SSS from the orange and blue lines overlap due to our projection, and four gray points lie on the top plane, again two points are overlapping, and one is out of the bounds of the graph. (D) Bifurcation diagram drawn with another different angle to emphasize differences in x-locations of each SSS. Three planes drawn at x = 0.1, 1, and 5. Four gray points lie on the left plane, with two overlapping, and one out of the bounds, four red points lie on the middle plane, with two overlapping, and two gray and two red points lie on the right plane. (PDF) [file pone.0102873.s002.pdf]

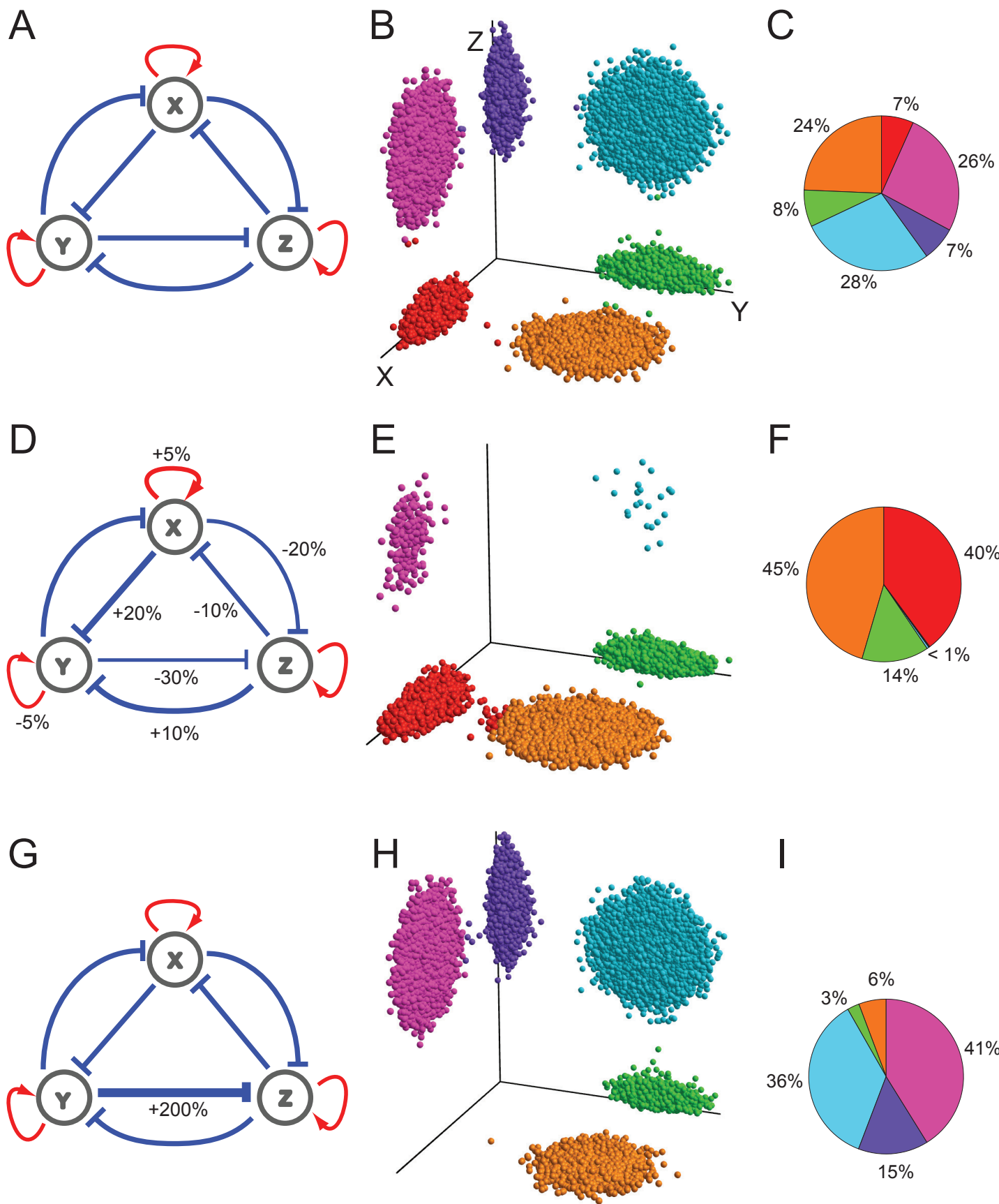

Figure S3  
Faucon *et al.*

Supplement: Figure S3 — Perturbations to network regulatory strengths affect the probability distribution of network states. Regulatory strengths for Network 84 were perturbed and the corresponding state probability distributions were generated using results of stochastic simulation. (A)–(C) Parameter strengths are identical to the green box in Figure 4, with simulation state plotted as point collections in B, and presented as a pie chart in C. (D)–(F) Random small perturbations to the regulatory strengths and corresponding state probability distributions. (G)–(I) Doubling in the strength of Y's inhibition of Z and corresponding state probability distributions. The magnitudes of perturbations are labeled on each network edge and are also represented by edge thickness. Axis labels of E and H are the same as those of B. Simulations for these distributions were calculated in the presence of a noise strength equal to 1, and each perturbation was simulated ten times. The point collections are color-coded based on their closest stable steady state, and their probabilities are summarized in panels C, F and I, respectively. (PDF) [file pone.0102873.s003.pdf]

A

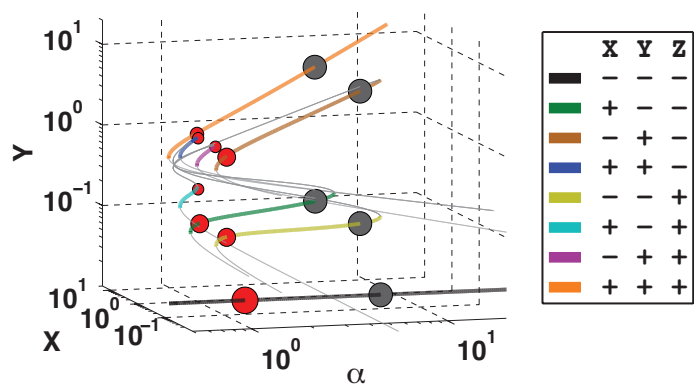

C

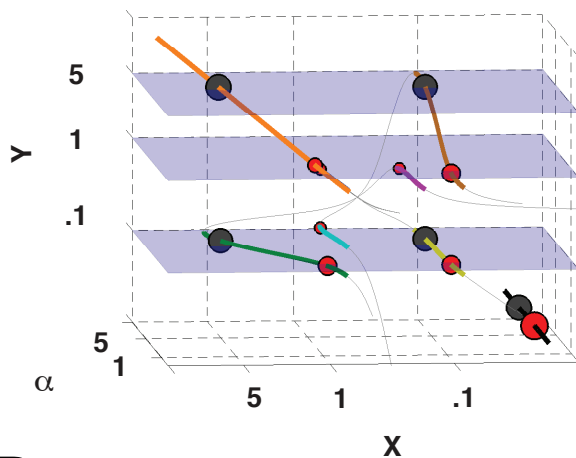

B

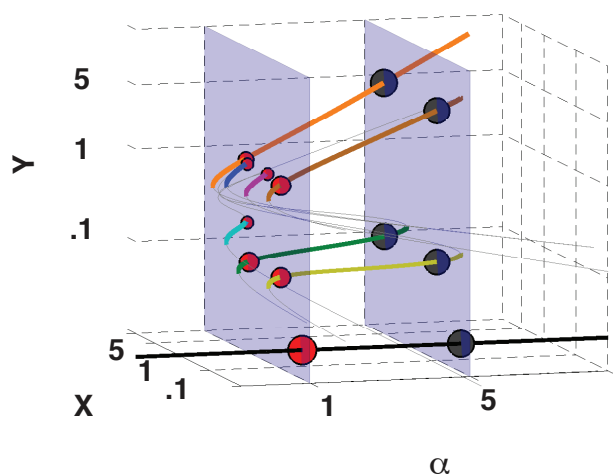

D

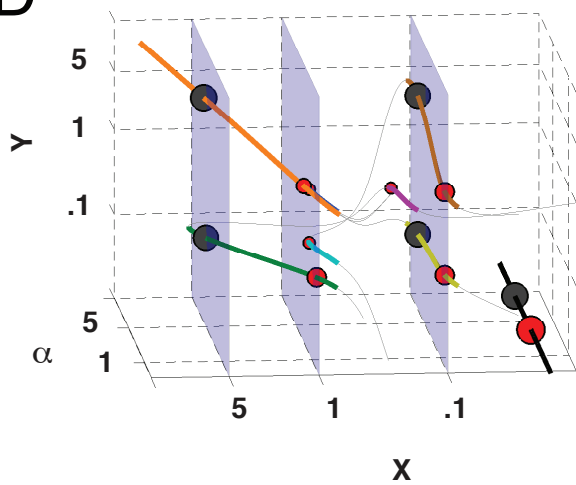

Figure S4  
Faucon *et al.*

Supplement: Figure S4 — Bifurcation diagram in Figure 5B shown with different viewing angles and visual assistance. (A) The bifurcation diagram of Figure5B is illustrated again to provide a frame of reference; the figure legend is consistent among all subfigures. The position of the all-OFF states have been shifted due to the logarithmic graphing, and that the all-OFF state remains at 0 concentration of x,y, and z regardless of alpha. (B) Bifurcation diagram is shown from the same angle as in A with planes drawn at α = 1 and α = 5 where the red and gray points lie precisely on the plane. (C) Bifurcation diagram drawn with a different angle to emphasize differences in y-locations of each SSS. Three planes drawn at y = 0.1, 1, and 5. Two gray points and three red points lie on the bottom plane, four red points lie on the middle plane, the SSS from the orange and blue lines overlap due to our projection, and two gray points lie on the top plane. (D) Bifurcation diagram drawn with another different angle to emphasize differences in x-locations of each SSS. Three planes drawn at x = 0.1, 1, and 5. Two gray points lie on the left plane, four red points lie on the middle plane, with two overlapping, and two gray and two red points lie on the right plane. (PDF) [file pone.0102873.s004.pdf]

A

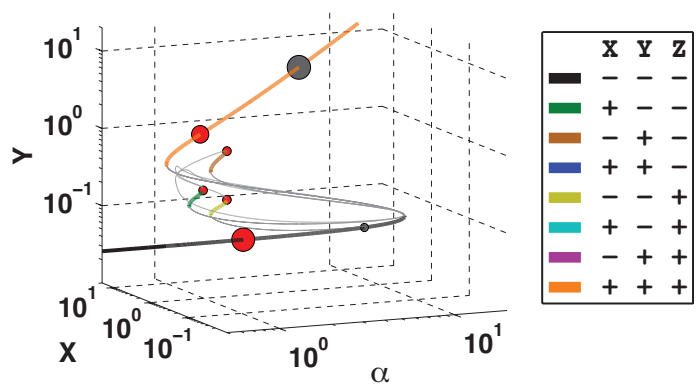

C

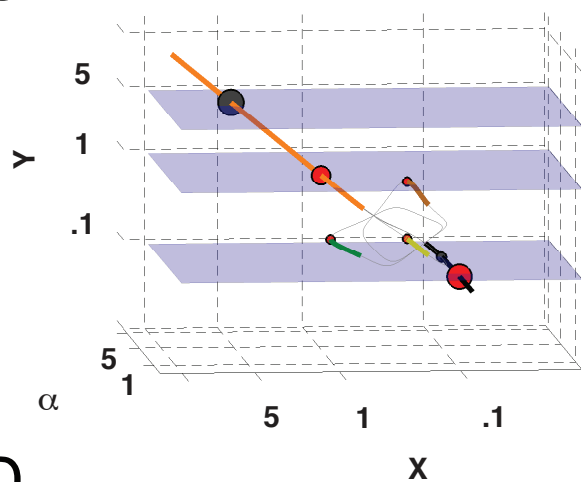

B

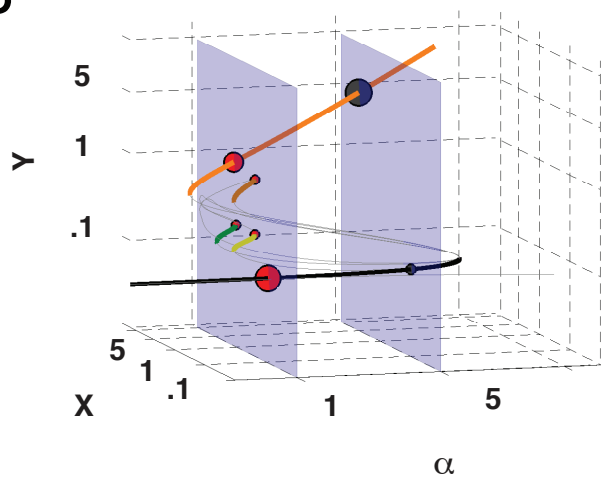

D

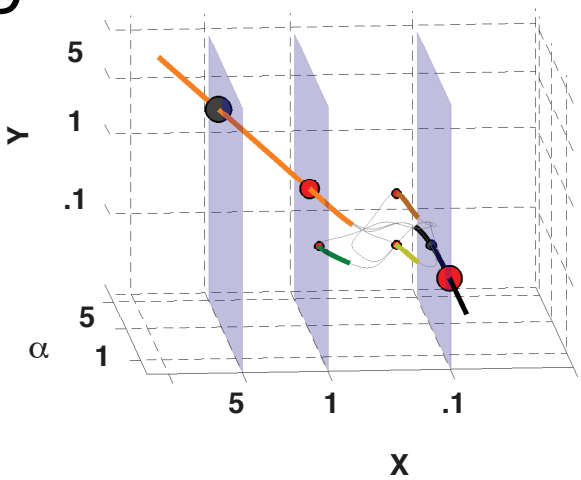

Figure S5  
Faucon *et al.*

Supplement: Figure S5 — Bifurcation diagram in Figure 7B shown with different viewing angles and visual assistance. (A) The bifurcation diagram of Figure7B is illustrated again to provide a frame of reference; the figure legend is consistent among all subfigures. (B) Bifurcation diagram is shown from the same angle as in A with planes drawn at α = 1 and α = 5 where the red and gray points lie precisely on the plane. (C) Bifurcation diagram drawn with a different angle to emphasize differences in y-locations of each SSS. Three planes drawn at y = 0.1, 1, and 5. One gray point and one red point lie on the bottom plane, two red points exist between the lower and middle planes (approximately 0.3), two red points lie on the middle plane, and one gray point lies on the top plane. (D) Bifurcation diagram drawn with another different angle to emphasize differences in x-locations of each SSS. Three planes drawn at x = 0.1, 1, and 5. One gray point lies on the left plane, two red points lie on the middle plane, two red points exist between the middle and right planes and one gray point and one red point lie on the right plane. (PDF) [file pone.0102873.s005.pdf]

A

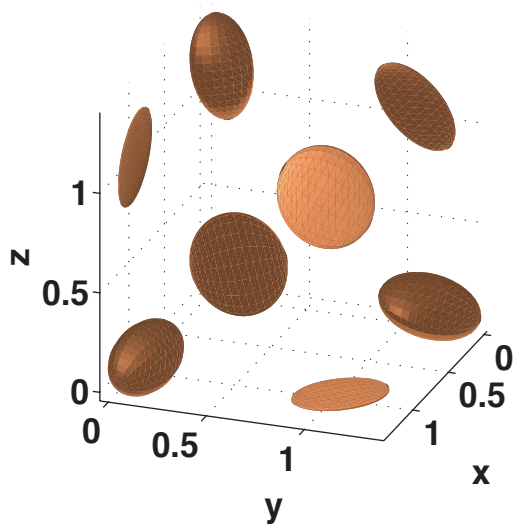

B

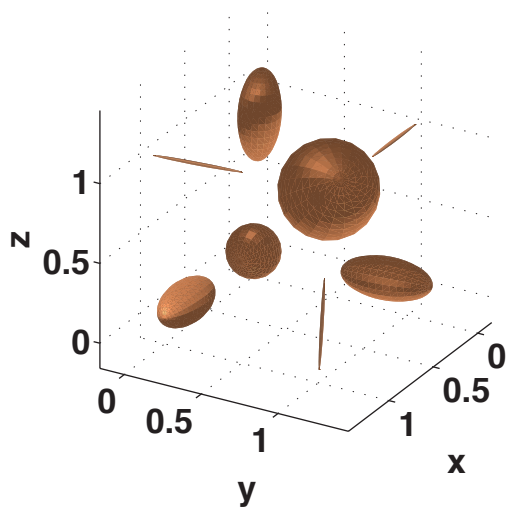

Figure S6  
Faucon *et al.*

Supplement: Figure S6 — Visual representation of constrained steady state sub-space under noise perturbations. (A) Trajectory clouds displayed in Figures 3C are approximated by 3D ellipsoids. The radius in each dimension is correlated with the inverse of the strength of the eigenvalue corresponding to a particular eigenvector. i.e. if the eigenvalue for an eigenvector is only weakly negative, then the cloud will be large in the direction of that eigenvector. Here the all-OFF cloud is scaled down in size manually so that it fits on the graph. The all-OFF and all-ON states are both disk-shaped, with their primary eigenvalue being significantly stronger and facing [1,1,1]. (B) Similarly, 3D ellipsoids were used to visualize possible cloud shape shown in Figure 5C. Different scaling parameters are used due to 1-ON and 2-ON states having a large difference in eigenvalue strength. Even with the modified parameters the 2-ON states are barely visible in the graph. This is consistent with stochastic simulation results shown in Figure 5C, where the trajectories barely spend any time in any of the 2-ON states. (PDF) [file pone.0102873.s006.pdf]
