# Supplementary material for: Gene Networks of Fully Connected Triads with Complete Auto-Activation Enable Multistability and Stepwise Stochastic Transitions
Source: PLoS One. 2014 Jul 24;9(7):e102873. doi: 10.1371/journal.pone.0102873 (PMC4109943; doi:10.1371/journal.pone.0102873)
Supplement: Text S1 — Supplementary texts of Materials and Methods. (DOCX) [file pone.0102873.s010.docx]

**SUPPORTING INFORMATION**

**1. Elimination of Redundant Networks**

To facilitate removal of duplicate topologies, each of the possible 2^9^=512 FCT configurations were first abstracted as a 3 x 3 connection matrix. Figure S1 shows examples of two possible FCTs and their respective connection matrices. In figure S1 the plus and minus signs indicate positive and negative regulation respectively. Despite the two FCT topologies in this example appearing to be superficially unique, switching of the Y and Z labels showed that they were in fact equivalent permutations of each other. Since all three nodes are meant to represent generic transcription factors and their exact order does not change the conclusion, permutations of networks are considered equivalent and only one of them was kept for further analysis.

I=$\left[ \begin{matrix} 1 & 0 & 0 \\ 0 & 1 & 0 \\ 0 & 0 & 1 \end{matrix} \right]$ P1=$\left[ \begin{matrix} 1 & 0 & 0 \\ 0 & 0 & 1 \\ 0 & 1 & 0 \end{matrix} \right]$ P2=$\left[ \begin{matrix} 0 & 1 & 0 \\ 1 & 0 & 0 \\ 0 & 0 & 1 \end{matrix} \right]$

P3=$\left[ \begin{matrix} 0 & 1 & 0 \\ 0 & 0 & 1 \\ 1 & 0 & 0 \end{matrix} \right]$ P4=$\left[ \begin{matrix} 0 & 0 & 1 \\ 0 & 1 & 0 \\ 1 & 0 & 0 \end{matrix} \right]$ P5=$\left[ \begin{matrix} 0 & 0 & 1 \\ 1 & 0 & 0 \\ 0 & 1 & 0 \end{matrix} \right]$

There were five possible permutation matrices in total for our analysis; P1-P5 above, with the identity matrix included for comparison. These permutation matrices account for all possible row and column switching, including three pair-wise switching, one clockwise rotation and one counter clockwise rotation of node labels. A single connection matrix, **M**, represented each FCT and each permutation matrix was denoted as **P**. If any two **M**s satisfied the equation M_1_=P_i_*M_2_*P_i_’ (*i*=1,2,3,4,5), where i represents any permutation matrix, then the two FCTs were considered a permutation of each other and one was eliminated. Enumerating each of the possibilities yielded 512 FCTs, after applying the permutation reduction 104 distinct FCTs remained as unique candidate topologies.

**2. ODE Modeling and High-Throughput Screening**

All networks are modeled from a three-dimensional ODE:

$\frac{dx}{dt}=a_{1}f_{1}(x) +b_{1}g_{1}(y)+c_{1}h_{1}(z)-\delta x$ (1)

$\frac{dy}{dt}=a_{2}f_{2}(x) +b_{2}g_{2}(y)+c_{2}h_{2}(z)-\delta y$ (2)

$\frac{dz}{dt}=a_{3}f_{3}(x) +b_{3}g_{3}(y)+c_{3}h_{3}(z)-\delta z$ (3)

In this formulation each variable (*x*,*y*,*z*) represents the protein abundance of one gene product (Equations 1-3). Each function (*f*, *g* or *h*) has the form F^n^ / (k^n^ + F^n^) when representing activation, and the form k^n^/(k^n^+F^n^) when representing inhibition, where *F* can be either *x*, *y*, or *z*. Network 84 shown in Figure 3A can be described by the ODE:

$\frac{dx}{dt}=a_{1}\frac{x^{n}}{k^{n}+x^{n}} +b_{1}\frac{k^{n}}{k^{n}+y^{n}}+c_{1}\frac{k^{n}}{k^{n}+z^{n}}-\delta x$ (4)

$\frac{dy}{dt}=a_{2}\frac{k^{n}}{k^{n}+x^{n}} +b_{2}\frac{y^{n}}{k^{n}+y^{n}}+c_{2}\frac{k^{n}}{k^{n}+z^{n}}-\delta y$ (5)

$\frac{dz}{dt}=a_{3}\frac{k^{n}}{k^{n}+x^{n}} +b_{3}\frac{k^{n}}{k^{n}+y^{n}}+c_{3}\frac{z^{n}}{k^{n}+z^{n}}-\delta z$ (6)

Network 1 shown in Figure 5A can be described by the ODEs:

$\frac{dx}{dt}=a_{1}\frac{x^{n}}{k^{n}+x^{n}} +b_{1}\frac{y^{n}}{k^{n}+y^{n}}+c_{1}\frac{z^{n}}{k^{n}+z^{n}}-\delta x$ (7)

$\frac{dy}{dt}=a_{2}\frac{x^{n}}{k^{n}+x^{n}} +b_{2}\frac{y^{n}}{k^{n}+y^{n}}+c_{2}\frac{z^{n}}{k^{n}+z^{n}}-\delta y$ (8)

$\frac{dz}{dt}=a_{3}\frac{x^{n}}{k^{n}+x^{n}} +b_{3}\frac{y^{n}}{k^{n}+y^{n}}+c_{3}\frac{z^{n}}{k^{n}+z^{n}}-\delta z$ (9)

The parameters (*a*_1-3_, *b*_1-3_, *c*_1-3_) represent the strengths of mutual or auto-regulation, be it activation or inhibition. We chose 421,875 parameter sets for all 104 networks to measure their probability of multistability. We defined a parameter set as multistable if it yields at least 5 stable steady states for a given network. This division was chosen to maximize the clarity in differentiating the multistable networks; relaxation of the requirement for multistability reduces the apparent differences in network stabilities. For mutual regulation strength, the parameters (*b*_1_, *c*_1_, *a*_2_, *c*_2_, *a*_3_, *b*_3_) were set to 0.1, 0.2, 0.5, 1 or 5; for auto-regulation strength, the parameters (*a*_1_, *b*_2_, *c*_3_) were set to 0.1, 0.5 or 1. Therefore, there were 5^6^*3^3^=421,875 parameter sets.

Network 1 with constitutive basal expression, shown in Figure 7A, can be described by the ODEs:

$\frac{dx}{dt}=\gamma_{1}+a_{1}\frac{x^{n}}{k^{n}+x^{n}} +b_{1}\frac{y^{n}}{k^{n}+y^{n}}+c_{1}\frac{z^{n}}{k^{n}+z^{n}}-\delta x$ (10)

$\frac{dy}{dt}=\gamma_{2}+a_{2}\frac{x^{n}}{k^{n}+x^{n}} +b_{2}\frac{y^{n}}{k^{n}+y^{n}}+c_{2}\frac{z^{n}}{k^{n}+z^{n}}-\delta y$ (11)

$\frac{dz}{dt}=\gamma_{3}+a_{3}\frac{x^{n}}{k^{n}+x^{n}} +b_{3}\frac{y^{n}}{k^{n}+y^{n}}+c_{3}\frac{z^{n}}{k^{n}+z^{n}}-\delta z$ (12)

Where previously mentioned parameters are identical and γ*_i ­_*(*i* = 1, 2, or 3) were set to 0.09 to introduce constitutive basal production to all three genes. As most other parameters had values <1, the γ value was selected to be large enough to mimic the overexpression of transcription factors in iPS experiments.

**3. Parameter Modulation and Alternative Models**

Choice of a particular functional form in a model can lead to a bias in observations, to counterbalance these effects multiple hill coefficients were examined, and alternate model forms were tested on networks 1 and 84. Utilizing the form described above (equations 7-12), hill coefficients of n=2 and n=3 were tested on networks 1 and 84. It is generally accepted that decreasing the hill coefficient, and therefore the nonlinearity of the equation, will reduce the range of multistable regions. This assumption was verified, by analysis of differences in stable steady state quantities across all parameter combinations. This loss can be counterbalanced through the decrease of mutual regulatory strength of the nodes, and the system will again be able to recover many of the stable steady states. This implies that increased nonlinearity in gene networks leads to an increase in stability, and/or higher levels of mutual regulatory strength.

The general trends in steady state stabilities are maintained with hill coefficients n=3 and n=2, while these coefficients lose approximately 9% and 30% of the stable states when compared with n=4. Importantly, we find a gradual decrease in multistability as n decreases from 4 to 2. As an alternative approximation metric, ~50% of n=4 parameters yield more than 1 stable steady state, ~44% of n=3 yield more than 1 stable steady state, and only 19% of n=2 parameters yield more than 1 stable steady state.

In addition to verification of alternate hill coefficients we also interrogated an alternate functional form to ensure that observations were not simply artifacts of the model form. In specific we modify the denominator of each term such that they are influenced by all other terms. The formula for network 84 under this modified functional form can be described by the ODEs:

$\frac{dx}{dt}=a_{1}\frac{x^{n}}{3*k^{n}+x^{n}+y^{n}+z^{n}} +b_{1}\frac{k^{n}}{3*k^{n}+x^{n}+y^{n}+z^{n}}+c_{1}\frac{k^{n}}{3*k^{n}+x^{n}+y^{n}+z^{n}}-\delta x$ (13)

$\frac{dy}{dt}=a_{2}\frac{k^{n}}{3*k^{n}+x^{n}+y^{n}+z^{n}} +b_{2}\frac{y^{n}}{3*k^{n}+x^{n}+y^{n}+z^{n}}+c_{2}\frac{k^{n}}{3*k^{n}+x^{n}+y^{n}+z^{n}}-\delta y$ (14)

$\frac{dz}{dt}=a_{3}\frac{k^{n}}{3*k^{n}+x^{n}+y^{n}+z^{n}} +b_{3}\frac{k^{n}}{3*k^{n}+x^{n}+y^{n}+z^{n}}+c_{3}\frac{z^{n}}{3*k^{n}+x^{n}+y^{n}+z^{n}}-\delta z$ (15)

Network 1 with modified functional form can be described by the ODEs:

$\frac{dx}{dt}=a_{1}\frac{x^{n}}{3*k^{n}+x^{n}+y^{n}+z^{n}} +b_{1}\frac{y^{n}}{3*k^{n}+x^{n}+y^{n}+z^{n}}+c_{1}\frac{z^{n}}{3*k^{n}+x^{n}+y^{n}+z^{n}}-\delta x$ (16)

$\frac{dy}{dt}=a_{2}\frac{x^{n}}{3*k^{n}+x^{n}+y^{n}+z^{n}} +b_{2}\frac{y^{n}}{3*k^{n}+x^{n}+y^{n}+z^{n}}+c_{2}\frac{z^{n}}{3*k^{n}+x^{n}+y^{n}+z^{n}}-\delta y$ (17)

$\frac{dz}{dt}=a_{3}\frac{x^{n}}{3*k^{n}+x^{n}+y^{n}+z^{n}} +b_{3}\frac{y^{n}}{3*k^{n}+x^{n}+y^{n}+z^{n}}+c_{3}\frac{z^{n}}{3*k^{n}+x^{n}+y^{n}+z^{n}}-\delta z$ (18)

We found that even under the modified functional form we maintain multistability.  Specifically both network 84 and network 1 achieve 2 or more stable steady states for over 75% of the parameter combinations; additionally network 84 achieves 3 stable steady states in over 32% of the parameter combinations tested, and network 1 achieves 3 stable steady states in over 20%. The revised model tends to require higher parameter values for a, b, and c, which is expected due to the increased magnitude of the denominator.

The alternate model supports a maximum of 5 stable steady states for network 1, and 4 in network 84.  The reason for this appears to be that the suggested functional form lacks the stabilization afforded by auto-activation.  Specifically, the auto-activation terms have a built-in mutual inhibition from the other network nodes.  This built-in inhibition causes the 2-ON states to be unstable, while causing the 3-ON states to require a mutual stabilization force. For example, in the case of the unstable 2-ON states, such as X-ON and Y-ON, a small increase in the expression level of X yields a further increase in X, eventually leading X to completely overpower Y.  This effect is not present in the original model due to the majority of the activating force on a node coming from its auto-activation (which is not intrinsically a function of other nodes).  In the case of the 3-ON state it is unattainable in the network 84 configuration for the same reason the 2-ON state is unattainable, however in network 1 high levels of activation (mutual and auto) allow for the stability of the 3-ON state. In general, our main conclusion regarding high possibility of multistability for these topologies remains largely true for this modified functional form, despite differences on exact number of SSS that the system could support.

**4. Screening Algorithm**

Identifying all possible stable steady states of a dynamical system is computationally intensive and typically requires sophisticated continuation algorithms and constant human interaction [1]. To achieve this goal, we numerically solved for the root of the right-hand side of each ODE set with over 1000 different initial guesses uniformly distributed over the entire state space. These solutions were then plugged into the Jacobian matrix and the eigenvalues were calculated. Solutions with all negative eigenvalues are SSS [2]. These SSS were then consolidated to remove duplicates.

The maximal of the initial guesses for each gene product is the corresponding activation rate divided by the degradation rate, which is the highest possible value of steady state abundance. For example, the maximal value of initial guesses for *x* is (*a1+b1+c1)/𝛿*. The initial guesses are divided into 11 segments ranging from [0-max] for each gene and therefore there are 11^3=1331 initial guesses. Since the maximal value of the initial guesses is the upper bounds of the system, it is ensured that no solutions are missing due to the range of the initial guesses. In addition, each network could theoretically have maximally 27 roots to the right hand side of the ODEs, which is very small compared to the number of initial guesses used. For this screen to miss a steady state, the initial guesses that lead to this state must occupy a volume smaller than 0.075% of the volume of the total state space. Root finding was applied with finer grids of initial guesses to networks 1 and 84 for comparison. These finer grids resulted in a small increase in the numbers of stable steady states found, but did not change the ranking or conclusions of Figure 2B. We found that 1331 initial guesses strikes a reasonable balance; the results are asymptotically approaching the true probability, and the computation time is not prohibitive.

To measure the probability of multistability for each FCT, this process was repeated with all 421,875 parameter sets. A parallel algorithm was utilized to distribute this task as an ensemble of independent computations. The workload was then distributed to a large computing cluster of over 200 processing units that was able to effectively utilize the parallelism. Parameter sets that gave rise to at least four stable steady states were counted as multistable. The multistability probability was then defined as the total number of multistable parameter sets counted divided by the total number of parameter sets, 421,875.

The algorithm we developed to screen for multistability can be summarized by the pseudo code below:

for each ODE network n

for each parameter set p

for each initial condition i

 root(n,p,i) = fsolve (eq = ODE n rhs,

par = parameter set p,

x0 = initial guess i);

end

u_root(n,p,1..r) = r consolidated unique roots;

for each unique root u

% stable steady states are defined as having

% all negative jacobian eigenvalues

jac = jacobian matrix for (n,p,u)

eigs= real eigenvalues of jac matrix

if (sum(eigs<0) < 3)

remove(n,p,u)

end

end

end

end

Each of the 104 ODE files were compiled from the unique connection matrices generated previously. The root finder used is *fsolve*, a built-in MATLAB implementation of the Levenberg-Marquardt algorithm. *For* loops without a data dependency are easily parallelized from within MATLAB so the parallel computation occurred at this level; each task had a subset of the parameter space for either one or two networks. Parameter sets for each network were broken into 200 pieces such that each compute node would process 104 jobs. We developed all of the non-stochastic software in MATLAB, and this code has been uploaded to GitHub (<https://github.com/pfaucon/Fully_connected_triads>), where it is publicly available under the GNUGPLV2 license (approved by OSI).

**5. Parameter Space Analysis**

In-depth analysis of Networks 1 and 84 allowed five levels for each parameter edge, [0.1 0.5 1 2 6], yielding 5^9 = 1,953,125 possible PCs. The number of PCs to analyze can be reduced using each of the 5 permutation matrices above (due to networks 1 and 84 being identical under each permutation). After the solutions have been computed, the resultant stable steady states should be used to permute PCs such that the number of PCs yielding qualitatively identical stable steady states is maximized. For example if 2 PCs yield 5 stable steady states, both yielding all 1-ON SSS and 2 of the 2-ON SSS. If one PC finds XY-ON and XZ-ON, while the other finds YX-ON and YZ-ON then one should be permuted so that the output SSS are the same, and the PCs can be compared directly.

During analysis PCs that yield 5 or more SSS for network 1, and 6 or more SSS for network 84 were selected. The threshold of selecting PCs is increased for Network 84 because otherwise the number of PCs to analyze is too large to manage. In a manner similar to network permutations described above, PCs that are permutations of each other were consolidated. This procedure resulted in 22 PCs for Network 1, and 298 PCs for Network 84. We then used MATLAB to generate an adjacency matrix that can be visualized in Cytoscape to describe the parameter space. An adjacency matrix describes which PCs should be connected to one another, and as such we use it to assist in grouping similar PCs. Two PCs are defined as adjacent if they vary only in one parameter strength, and that parameter differs by only one increment of our parameter space (e.g. 1.0 vs 2.0, but not 0.1 vs 1.0). Cytoscape is then able to create a visualization of the adjacency matrix, and automatically lay out the graph. Specifically the built-in force-directed layout provided clear distinctions of the groups. Following this separation, the node groups were then manually analyzed to generate the FCT diagrams in Figures 4C and 6C.

**6. Bifurcation Analysis**

Bifurcation diagrams were generated using MatCont (<http://www.matcont.ugent.be/>), a continuation toolbox for MATLAB. Computation of a bifurcation diagram starts from a known stable steady state and continues to all the branches of the diagram [3]. For both network 1 and 84, the starting parameters chosen were all self-regulation at 1, all mutual regulation at .1. With these parameters both networks have 8 stable steady states, which are used as starting points for continuation. Bifurcation analysis was then performed on the parameter α, which represents the auto-activation strength for each node (α =a1=b2=c3). For network 1 the standard tolerance of 1E-4 resulted in numerical errors around the all-OFF state, therefore more strict tolerance of 1E-6 and decreased step size were used. These stricter settings were also used for network 84 for consistency. Custom scripts were used to automatically locate each branch point and continue the bifurcation diagram along each branch until no new branching points exist. Eigenvalues of the Jacobian matrices at each Hopf point were then used to distinguish between stable and unstable steady states, which were then color coded in Figures 3B, 5B, and 7B.

**7. Details for Stochastic Simulations**

stochastic simulations were carried out as outlined in the EXPERIMENTAL PROCEDURES.

To calculate the first passage time (FPT), the simulation was stopped once the abundance of chemical species satisfied the condition:

$(x-X_{s})^{2}+(y-Y_{s})^{2}+(z-Z_{s})^{2}<1000$ (19)

Where *x*, *y*, and *z* represent transcription factor abundances and *X_s_*, *Y_s_*, and *Z_s_* represent their abundances at the corresponding state. For Figure 8A, the simulation was initiated from the origin and *X_s_*, *Y_s_*, and *Z_s_* equal 126, 126, and 126 (all-ON state). For Figure 8B, the initial condition was 117, 11 and 11 (X-ON YZ-OFF state) and the values for *X_s_,Y_s_* and *Z_s_* were 11, 11 and 117 (Z-ON XY-OFF state).

**Supplemental References**

1. Ermentrout B (2002) Simulating, analyzing, and animating dynamical systems : a guide to XPPAUT for researchers and students. Philadelphia: Society for Industrial and Applied Mathematics. xiv, 290 p. p.

2. Strogatz SH (1994) Nonlinear Dynamics and Chaos: With Applications to Physics, Biology, Chemistry, and Engineering. Reading, Mass.: Addison-Wesley Pub. xi, 498 p.

3. Dhooge A, Govaerts W, Kuznetsov YA, Meijer HGE, Sautois B (2008) New features of the software MatCont for bifurcation analysis of dynamical systems. Math Comput Model Dyn Syst 14: 147–175. doi:10.1080/13873950701742754.

**Supplementary Figure and Movie Legends**

**Figure S1. An example of the connection matrices and topological permutations used to identify unique FCTs**. In the two FCT connection matrices illustrated, each row represents the source of regulation and each column the recipient of regulation, with symbols representing the activation or repression. As indicated by the arrows, these two matrices are permutations of each other after switching the Y and Z labels. Complete permutation analysis involved both row and column switching.

**Movie S1. A movie showing the simulated temporal evolution of chemical species in Figure 3.** The temporal evolution of transcription factors is shown as an elongating blue ribbon. All conditions are the same as in the right panel of Figure 3C. Only the first 5000 time points were animated.

**Movie S2. A movie showing the simulated temporal evolution of chemical species in Figure 5.** The temporal evolution of transcription factors is shown as an elongating blue ribbon. All conditions are the same as in right panel of Figure 5C. Only the first 5000 time points are animated.

**Movie S3. A movie showing the simulated temporal evolution of chemical species in Figure 7.** The temporal evolution of transcription factors is shown as an elongating blue ribbon. All conditions are the same as in Figure 6A. Noise strength is 1.5. Only the first 200 time points are animated because the simulation equilibrated rapidly.

**Figure S2. Bifurcation diagram in Figure 3B shown with different viewing angles and visual assistance.** (A) The bifurcation diagram of Figure3B is illustrated again to provide a frame of reference; the figure legend is consistent among all subfigures. (B) Bifurcation diagram is shown from the same angle as in A with planes drawn at α =1 and α=5 where the red and gray points lie precisely on the plane. (C) Bifurcation diagram drawn with a different angle to emphasize differences in y-locations of each SSS. Three planes drawn at y=0.1, 1, and 5. Two gray points and two red points lie on the bottom plane, four red points lie on the middle plane, the SSS from the orange and blue lines overlap due to our projection, and four gray points lie on the top plane, again two points are overlapping, and one is out of the bounds of the graph. (D) Bifurcation diagram drawn with another different angle to emphasize differences in x-locations of each SSS. Three planes drawn at x=0.1, 1, and 5. Four gray points lie on the left plane, with two overlapping, and one out of the bounds, four red points lie on the middle plane, with two overlapping, and two gray and two red points lie on the right plane.

**Figure S3. Bifurcation diagram in Figure 5B shown with different viewing angles and visual assistance.** (A) The bifurcation diagram of Figure5B is illustrated again to provide a frame of reference; the figure legend is consistent among all subfigures. The position of the all-OFF states have been shifted due to the logarithmic graphing, and that the all-OFF state remains at 0 concentration of x,y, and z regardless of alpha. (B) Bifurcation diagram is shown from the same angle as in A with planes drawn at α =1 and α=5 where the red and gray points lie precisely on the plane. (C) Bifurcation diagram drawn with a different angle to emphasize differences in y-locations of each SSS. Three planes drawn at y=0.1, 1, and 5. Two gray points and three red points lie on the bottom plane, four red points lie on the middle plane, the SSS from the orange and blue lines overlap due to our projection, and two gray points lie on the top plane. (D) Bifurcation diagram drawn with another different angle to emphasize differences in x-locations of each SSS. Three planes drawn at x=0.1, 1, and 5. Two gray points lie on the left plane, four red points lie on the middle plane, with two overlapping, and two gray and two red points lie on the right plane.

**Figure S4. Bifurcation diagram in Figure 7B shown with different viewing angles and visual assistance.** (A) The bifurcation diagram of Figure7B is illustrated again to provide a frame of reference; the figure legend is consistent among all subfigures. (B) Bifurcation diagram is shown from the same angle as in A with planes drawn at α =1 and α=5 where the red and gray points lie precisely on the plane. (C) Bifurcation diagram drawn with a different angle to emphasize differences in y-locations of each SSS. Three planes drawn at y=0.1, 1, and 5. One gray point and one red point lie on the bottom plane, two red points exist between the lower and middle planes (approximately 0.3), two red points lie on the middle plane, and one gray point lies on the top plane. (D) Bifurcation diagram drawn with another different angle to emphasize differences in x-locations of each SSS. Three planes drawn at x=0.1, 1, and 5. One gray point lies on the left plane, two red points lie on the middle plane, two red points exist between the middle and right planes and one gray point and one red point lie on the right plane.

**Figure S5. Visual representation of constrained steady state sub-space under noise perturbation.** These figures represent the trajectory clouds displayed by figures 3C (A) and 5C (B). The shape of the clouds is correlated with the inverse of the strength of the eigenvalue corresponding to a particular eigenvector. i.e. if the eigenvalue for an eigenvector is only weakly negative, then the cloud will be large in the direction of that eigenvector. In (A) the all-OFF cloud is scaled down in size manually so that it fits on the graph. The all-OFF and all-ON states are both disk-shaped, with their primary eigenvalue being significantly stronger and facing [1,1,1] In (B) different scaling parameters are used due to 1-ON and 2-ON states having a large difference in eigenvalue strength. Even with the modified parameters the 2-ON states are barely visible in the graph. Because of the eigenvalue normalization step used, the 1-ON states are visible and apparently have a larger cloud, however the eigenvalues of the 1-ON state are significantly more negative (stable) than the 2-ON states.

**Figure S6. Perturbations to network regulatory strengths affect the probability distribution of network states.** Regulatory strengths for Network 84 were perturbed and the corresponding state probability distributions were generated by simulation. (A)- (C) Identical strengths as those used in Figure 3, with the associated state probability distributions plotted as point collections in B and pie chart in C. (D)-(F) Random small perturbations to the regulatory strengths and corresponding state probability distributions. (G)-(I) Doubling in the strength of Y’s inhibition of Z and corresponding state probability distributions. The magnitudes of perturbations are labeled on each network edge and are also represented by edge thickness. Axis labels of E and H are the same as those of B. Simulations for these distributions were calculated in the presence of a noise strength equal to 1, and each perturbation was simulated ten times. The point collections are color- coded based on their vicinity to the corresponding states, and their probabilities are summarized in panels C, F and I, respectively.
